# Toward an idiomatic framework for cognitive robotics

**DOI:** 10.1016/j.patter.2022.100533

**Published:** 2022-07-08

**Authors:** Malte Rørmose Damgaard, Rasmus Pedersen, Thomas Bak

**Affiliations:** 1 Department of Electronic Systems, Automation and Control, Aalborg University, 9220 Aalborg, Denmark

**Keywords:** robotics, cognitive robotics, cognitive architecture, framework, probabilistic programming, probabilistic programming language, probabilistic inference, variational inference, message-passing algorithm, stochastic variational inference, probabilistic graphical model

## Abstract

Inspired by the “cognitive hourglass” model presented by the researchers behind the cognitive architecture called Sigma, we propose a framework for developing cognitive architectures for cognitive robotics. The main purpose of the proposed framework is to ease development of cognitive architectures by encouraging cooperation and re-use of existing results. This is done by proposing a framework dividing development of cognitive architectures into a series of layers that can be considered partly in isolation, some of which directly relate to other research fields. Finally, we introduce and review some topics essential for the proposed framework. We also outline a set of applications.

## Highlights

• The proposed framework divides development of cognitive architectures into layers
• The framework spans the best-known approaches employed within cognitive robotics
• The framework is centered around modern probabilistic programming techniques
• Two applications demonstrate the concepts of the framework

## The bigger picture

For many decades, robots have been expected to transfigure the world we live in, and in many ways they already have, by increasingly taking over dull, dirty, and dangerous jobs. However, for robots to integrate fully and seamlessly into human societies, robots need to be able to learn and reason from experience and effectively deal with unpredictable and dynamic environments. Developing robotic systems with such intelligence is a tremendous and difficult task, which has led to the foundation of the new multi-disciplinary scientific field called cognitive robotics, merging research in adaptive robotics, cognitive science, and artificial intelligence. To ease merging research from these scientific fields, we propose a general framework for developing intelligent robotic systems based on recent advancements in the machine learning community. We hope that this framework will aid researchers and practitioners in bringing even more helpful robots into our societies.

## Graphical abstract

**Figure:**
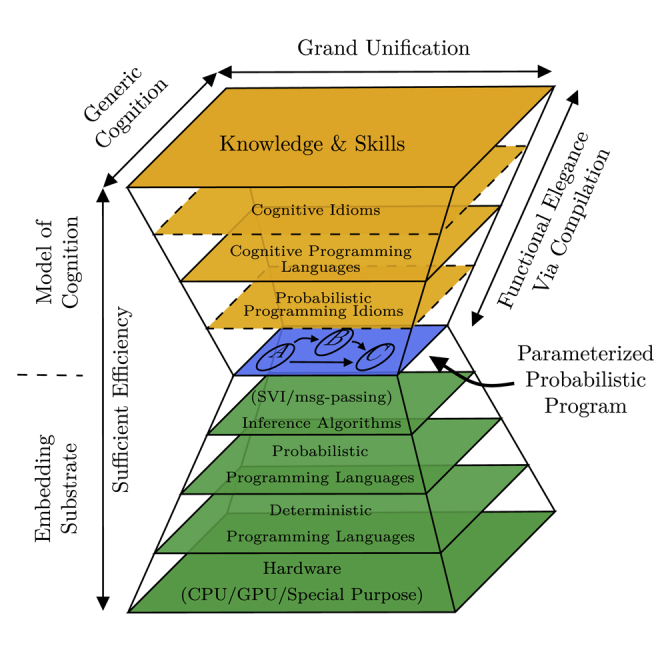

## Teaser

Damgaard et al. propose a general framework for developing cognitive architectures for robots. The proposed framework divides development into a series of layers separating models of cognition from the algorithms that implement them. Based on modern probabilistic programming and variational inference algorithms, the proposed framework aims to support the four desiderata generic cognition, grand unification, functional elegance, and sufficient efficiency. The concepts of the framework are demonstrated through two robotic applications.

## Introduction

Research in cognitive robotics originates from a need to perform and automate tasks in dynamic environments and with close or direct interaction with humans. Uncertainty about the environment and complexity of the tasks require robots with the ability to reason and plan while being reactive to changes in their environment. To achieve such behavior, robots cannot rely on predefined rules of behavior,^1^ and inspiration is taken from cognitive architectures.

Cognitive architectures provide a model for information processing that can capture robot functionalities. In combination with acquired sensory data, they can potentially generate intelligent autonomous behavior.^2^ Cognitive architectures dates back to the 1950s,^3^ with the grand goal of implementing a full working cognitive system.^4^ From this considerable challenge, an abundance of architectures has evolved, and a recent survey suggests that the number of existing architectures has reached several hundred.^3^ Some are aimed toward robotics applications; e.g., Robo-Soar,^5^ CARACaS,^6^ and RoboCog.^7^ Unfortunately, most of these architectures take wildly different approaches to model cognition and are implemented in different programming languages. Most of these architectures are constructed from a diverse set of specialized modules, making it difficult to expand, combine, and re-use parts of these architectures. In fact, this could be one of the contributing reasons for the abundance of architectures. The authors of a recent study of cognitive architectures related to the iCub robot explain their decision to start from scratch rather than relying on existing architectures: “this decision was made in order to gain more freedom for future expansions of the architecture.”^8^ In other words, despite the abundance of architectures, existing architectures were not deemed flexible enough to build upon. Following the arguments for developing an interface layer for artificial intelligence put forward by other researchers,^9^ we argue that a unifying and standardized framework for developing new cognitive architectures aimed at cognitive robotics could potentially remedy these issues and ease development of cognitive robotics.

In recent years, a community consensus has emerged about a standard model of human-like minds; i.e., computational entities whose structures and processes are substantially similar to those found in human cognition.^10^ Although this “standard model of the mind” spans key aspects of structure and processing, memory and content, learning, and perception and motor, it is agnostic to the best practice for modeling and implementing these things.^10^

In line with the idea proposed by the researchers behind the cognitive architecture Sigma,^4^ we argue that the evolution of the scientific field of cognitive robotics could benefit from anchoring new implementations around a common theoretical elegant base separating a specific model of a part of cognition from the algorithm that implements it. This theoretical base could allow new functionalities to evolve hierarchically just like software libraries build on top of each other, allowing discussions and development to flourish at different levels of abstractions and enable synergy with other research fields.

To explain the cognitive architecture Sigma,^4^ the authors present a cognitive hourglass model based on the following four desiderata:

• grand unification, spanning all of cognition;
• generic cognition, spanning natural and artificial cognition;
• functional elegance, achieving generically cognitive grand unification with simplicity and theoretical elegance; and
• sufficient efficiency, efficient enough to support the anticipated uses in real time.

Although grand unification and sufficient efficiency aligns well with the needs of cognitive robotics, the need for generic cognition and functional elegance is subtle for cognitive robotics. Although the end goal of cognitive robotics might only be functional artificial intelligence, building on something that is potentially also able to model natural intelligence would allow artificial intelligence to more easily benefit from insights obtained by modeling natural intelligence and vice versa. Similarly, functional elegance is not a goal of cognitive robotics per se. Still, it could allow researchers and practitioners working on different levels of cognition to obtain a common reference point and understanding at a basic level, potentially easing cooperation and re-use of results and innovative ideas.

In an attempt to obtain all four of these desiderata, the so-called “graphical architecture” based on inference over probabilistic graphical models is placed at the waist of Sigma’s cognitive hourglass model, gluing everything together just like the internet protocol (IP) in the internet hourglass model.^11^ Functional elegance is obtained by recognizing and developing general architectural fragments and based on these defining idioms, which can be re-used in modeling different parts of cognition. Having defined sufficiently general idioms, the hope is to be able to develop full models of cognition from a limited set of such idioms and thereby achieve functional elegance while achieving the three other desiderata.^4^ With roots in the given desiderata, Sigma’s cognitive hourglass model, in many ways, could constitute a unifying and standardized framework for cognitive robotics. However, as we will elaborate under Sigma’s cognitive hourglass model, the model commits to specific architectural decisions, which hinders utilization of new technology and ideas; e.g., their commitment to the sum-product algorithm prevents use of new algorithms for efficient probabilistic inference. The benefits of utilizing probabilistic graphical models specifically for cognitive robotics have been corroborated in many studies. For example, learning and representing the hierarchical structure of concepts,^12^ simultaneous lexical and spatial concept acquisition,^13^ navigation utilizing the learned concepts,^14^ and the interaction between multiple probabilistic graphical models^15^ have been studied. This research has led to two frameworks, SERKET^16^ and its extension Neuro-SERKET,^17^ with the goal of connecting multiple probabilistic graphical models on a large scale to construct cognitive architectures for robotics. Being based solely on probabilistic graphical models, SERKET and Neuro-SERKET currently do not seem to incorporate logic, making it difficult to implement symbolic approaches in these frameworks. In fact, researchers behind the work related to Markov logic and the system called Alchemy have argued that the combination of logic, especially first order, and pure probabilistic graphical models is necessary to compose a sufficiently general interface layer between artificial intelligence and the algorithms that implement it.^9^ Similar to Sigma, models of cognition are implicitly tied to specific inference algorithms in Alchemy and the SERKET frameworks. Thus, these cannot be considered suitable as generalized frameworks.

Based on the observation that the layers of Sigma’s cognitive hourglass model can be divided conceptually into more generalized layers, we propose a generalized cognitive hourglass model based on recent advances within machine learning that makes no such commitments. More specifically, the main contribution of this paper is a framework for developing cognitive architectures for robotics centered around probabilistic programs that

• separates a specific model of cognition from the algorithm that implements it,
• allows the combination of logic and probabilistic models,
• is not tied to specific inference algorithms,
• provides a structure dividing development of cognitive architectures into layers, and
• embraces the same four desiderata as Sigma.

The presented generalized cognitive hourglass model is a flexible framework for guiding and discussing future development of cognitive robotics. We do not intend to construct a new specific cognitive architecture. Our framework should be viewed as a space of systems subsuming the Sigma, Alchemy, and SERKET frameworks, among others, and our intent with this framework and manuscript is

1. to provide a framework for other researchers to expand,
2. to ease development of cognitive architectures for robotics by encouraging and mitigating cooperation and re-use of existing results, and
3. to highlight some of the current state-of-the-art technology available to advance this research field.

Under Sigma’s cognitive hourglass model, we briefly introduce Sigma’s cognitive hourglass model in more detail. Based on this, we present our generalized cognitive hourglass model as a framework for developing new cognitive architectures aimed at cognitive robotics under Generalized cognitive hourglass model. Under Probabilistic programs, we provide a brief introduction to probabilistic programs because the presented framework is built around them. Explaining the functionality of probabilistic programs with conventional methods can be difficult; therefore, under Generative flow graphs, we present a graphical representation of probabilistic programs we call “generative flow graphs.” We do so in the hope that it will ease dissemination of new models of parts of cognition developed within the proposed framework. Being fundamental for achieving functional elegance within the proposed framework, we formally introduce the concept of probabilistic programming idioms under Probabilistic programming idioms and explain how “generative flow graphs” can aid identification of such idioms. Under Inference algorithms, we discuss the intrinsic problem of performing approximate inference in complex probabilistic programs and present some modern algorithms to tackle this problem for cognitive robotics. Because probabilistic programming languages form the foundation of the present framework, we provide a brief survey of probabilistic programming languages relevant to the framework under Probabilistic programming languages. Finally, under Application examples, we present some preliminary work to support the presented framework.

## Results

### Sigma’s cognitive hourglass model

**Figure 1:**
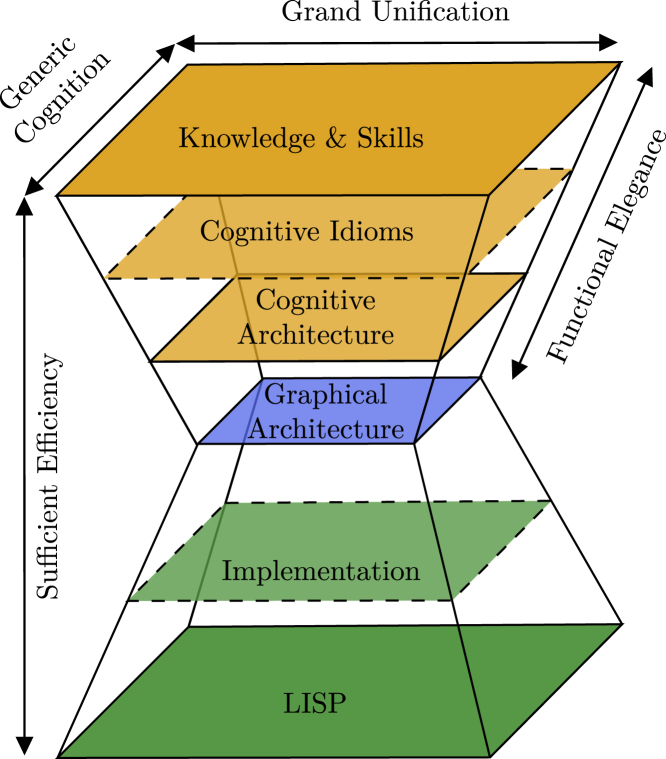
Sigma’s cognitive hourglass model Shown is a loose re-drawing of figures of the cognitive hourglass model presented in Rosenbloom et al.^4^ Layers with dashed borders are not recognized as distinguishable layers by Rosenbloom et al.^4^

Figure 1 illustrates how the dimensions of Sigma’s cognitive hourglass model relate to the four desiderata. The top layer of the hourglass represents all of the knowledge and skills implemented by the cognitive system. This includes high-level cognitive capabilities, such as reasoning, decision-making, and meta cognition, as well as low-level cognitive capabilities, such as perception, attention, and formation of knowledge and memory that could potentially be inspired by human cognition. But it also includes artificial cognitive capabilities such as, e.g., creation of grid maps common in robotics. Therefore, the extent of this layer corresponds to the achievable extent of grand unification and generic cognition.

The “cognitive architecture” layer defines central architectural decisions, such as utilization of the cognitive cycle and tri-level control structure for information processing, and division of memory into a perceptual buffer, working memory, and long-term memory. It also defines other architectural concepts, such as “functions,” “structures,” “affect/emotion,” “surprise,” and “attention.” Therefore, the cognitive architecture induces what can be considered a “cognitive programming language,” in which all of the knowledge and skills in the top layer can be embodied and learned. As an intermediate layer, cognitive idioms provide design patterns, libraries, and services that ease implementation of knowledge and skills.

Below the cognitive architecture and at the waist of the model is the graphical architecture constituting a small elegant core of functionality. Functional elegance is obtained by compilation of knowledge and skills through a series of layers into a common representation in the graphical architecture. This graphical architecture primarily consists of probabilistic inference over graphical models (more specifically, factor graphs) utilizing the sum-product algorithm^18^ plus the following extensions:

1. each variable node is allowed to correspond to one or more function variables,
2. special purpose factor nodes,
3. and the possibility of limiting the direction of influence along a link in the graph.

Of these extensions, the two first are merely special-purpose optimizations for the inference algorithm; i.e., a part of the implementation layer in Figure 1. According to the authors, the third extension has “a less clear status concerning factor graph semantics.”^4^ Finally, the graphical architecture is implemented in the programming language LISP. In this model, sufficient efficiency is achieved as the cumulative efficiency of all layers; i.e., an efficient implementation in LISP is futile when models of knowledge and skills are inefficient for a given task.

The model shown in Figure 1 commits to multiple, more or less restrictive decisions, such as utilization of factor graphs and the sum-product algorithm at its core, the “cognitive cycle,” the tri-level control structure, and LISP as the exclusive implementation language. Although these commitments may be suitable for the specific cognitive architecture Sigma mainly targeted human-like intelligence, they would hinder exploration of new ideas and utilization of new technologies, making this model less suitable as a general framework.

### Generalized cognitive hourglass model

Although Sigma’s cognitive hourglass model has an advantageous structure with roots in highly appropriate desiderata, it is not suitable as a general framework because of some exclusive structural commitments. We argue that these structural commitments are mostly artifacts of the limited expressibility of factor graphs and the sum-product algorithm.

Consider, for instance, the “cognitive cycle” dividing processing into an elaboration and adaption phase. The elaboration phase performs inference over the factor graph, and the adaption phase modifies the factor graph before further inference. We argue that this two-phase division of processing is caused by the need for the sum-product algorithm to operate on a static factor graph. This cognitive cycle makes the tri-level control structure necessary to make cognitive branching and recursion possible. Similarly, we argue that the third extension of the factor graph semantics employed in the cognitive architecture Sigma is nothing more than a simple control flow construct over the information flow in the graphical model and inference algorithm. It is easy to imagine how other control flow constructs, such as recursion, loops, and conditionals, could also be advantageous in modeling cognition.

Basically, we believe that special-purpose implementations of architectural constructs, such as the two-phase “cognitive cycle” employed by Sigma and similar cognitive architectures, have been necessary previously because of the limitations of the available modeling tools. The flexibility of probabilistic programs provided by the possibility of incorporating I/O operations, loops, branching, and recursion into a probabilistic model should instead permit representation of such constructs as probabilistic programming or cognitive idioms.

**Figure 2:**
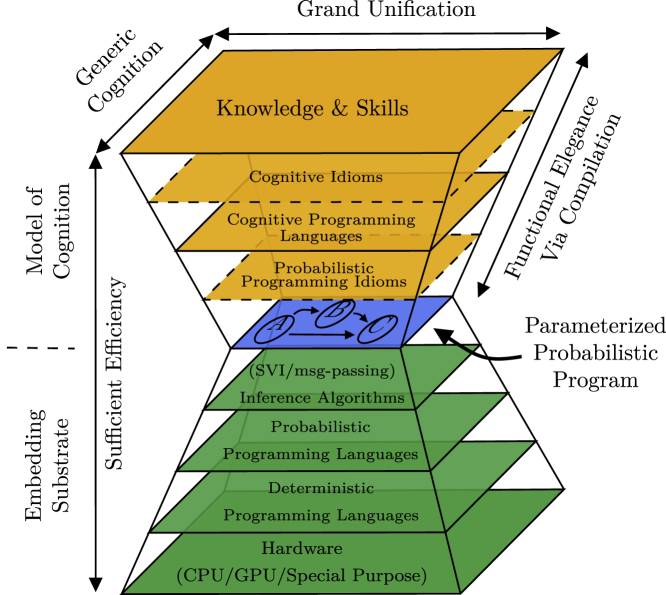
The generalized cognitive hourglass model Our proposal for a generalized cognitive hourglass model. Dashed borders indicates layers that are not necessarily recognized as distinguishable layers but could help in development of the other layers.

In Figure 2, we present our proposal for a more general cognitive hourglass model having probabilistic programs at its waist as the theoretical modeling base. Just like the model under Sigma’s cognitive hourglass model, our model is composed of a series of layers that expand away from the waist of the hourglass. On top of the pure probabilistic program, we might be able to recognize program fragments that are sufficiently general to be considered idioms. From these idioms, it might be possible to construct dedicated programming languages for expressing cognitive behavior, knowledge, and skills, such as the “cognitive language” employed in the cognitive architecture Sigma.^4^ In this framework, functional elegance above the probabilistic program is obtained via compilation of knowledge and skills through appropriate cognitive programming languages into probabilistic programs. Below the probabilistic programs different inference algorithms can carry out the necessary inference in the probabilistic program. Different versions of these inference algorithms can potentially be implemented in different probabilistic languages. The probabilistic program and probabilistic programming language can be situated in standard deterministic programming languages. One needs not even use the same deterministic programming language for both,^19^ separating development of models of cognition from development of the algorithms that implement them. Finally, the deterministic programming languages allow us to execute a model of cognition on different types of hardware doing the actual computations. When comparing Sigma’s hourglass model with the generalized hourglass model, the complexity might seem to have increased. However, this is not the case. The generalized hourglass model simply highlights some of the components implicit in Sigma’s hourglass model.

We expect that this model is sufficiently general to be considered a framework for research and development of cognitive robotics, and as stated in the Introduction, the presented model should be considered a space of systems subsuming others. Our model subsumes Sigma, which limits the probabilistic programs at the waist of our model to factor graphs and limits inference to the sum-product algorithm. As another example, consider the SERKET frameworks. In both frameworks, exact message-passing is used to perform inference on probabilistic graphical models with discrete and finite variables; otherwise, sampling importance resampling is used. Both of these frameworks can also be considered special cases of our framework, with “modules” and their connections somehow resembling what we have chosen to call “probabilistic programming idioms.” The “modules” in SERKET and Neuro-SERKET are supposed to be fully defined and self-contained. In contrast, our definition of “probabilistic programming idioms” allows nesting and, e.g., class definitions with abstract methods, as we will exemplify under Application examples. As a third example, Alchemy may also be considered one instance of our framework, limiting the probabilistic programs at the waist to Markov logic and utilizing a combination of Markov chain Monte Carlo and lifted belief propagation for inference.^9^ In fact, because probabilistic programs can be considered an extension of deterministic programs, it should even be possible to situate emergent, symbolic, and hybrid approaches to cognitive architectures in this framework, covering the full taxonomy considered in Kotseruba and Tsotsos.^3^ In our framework, constructs such as the cognitive cycle and tri-level control structure could potentially be expressed as probabilistic programming idioms rather than special-purpose architectural implementations. Similarly, incorporation of results from other research areas, such as deep learning, is only limited to the extent that a given probabilistic programming language and corresponding inference algorithms can incorporate essential tools used in these research areas; i.e., automatic differentiation for deep learning. This framework gives a satisfying view of the foundational hypothesis in artificial intelligence about substrate independence^10^ by cleanly separating the model of cognition (i.e., the probabilistic program and everything above it) from the organic or inorganic substrate on which it exists (i.e., everything below the probabilistic program).

Although the above might sound promising, the choice of probabilistic programs as a focal point also has important ramifications. In general, we cannot guarantee the existence of an analytic solution for all models, and even if a solution exists, it might be computationally intractable.^19^ Therefore, we have to endure approximate solutions. Though this might sound restrictive, this is also the case for most other complex real-world problems. In fact, it can be considered a form of bounded rationality consistent with the concept of “satisficing,” stating that an organism confronted with multiple goals does not have the senses or the wits to infer an “optimal” or perfect solution and, thus, will settle for the first solution permitting satisfaction at some specified level of all of its needs.^20^ The second important ramification is that the model with its roots in probabilistic and deterministic programming languages is only applicable to the extent to which the hypothesis that artificial cognition can be grounded in such programming languages is valid. However, this is currently a widely accepted hypothesis.

It is important to stress that the layers of the proposed framework are not independent. On the contrary, as the technological possibilities and community knowledge evolve, changes in one layer might open new possibilities in the layers above. Similarly, the need for new features in one layer might guide the research directions and development of the layers below. However, this structure is exactly what would allow further discussions and development in cognitive robotics to evolve at different levels of abstractions and benefit from other research fields related to the layers below probabilistic programs. In the layers above probabilistic programs, development and identification of probabilistic programming idioms, cognitive programming languages, and cognitive idioms mitigate cooperation and re-use of existing results. The framework thus minimizes the burden of developing new cognitive architectures by allowing researchers to focus their energy on specific layers, or parts thereof, in the hourglass model rather than dealing with all of the details of a cognitive architecture. The extent to which the burden of development is reduced thus depends on the technology available in each of the layers of the hourglass.

### Preliminaries

In this paper, we do not distinguish between probability density functions and probability mass functions and jointly denote them as probability functions. The symbol ∫ is used to denote integrals and summations depending on the context. In general, we use *z* to denote latent random variables, *x* to denote observed random variables, *p*(…) to denote “true” probability functions, *q*(…) to denote approximations to “true” probability functions, θ to denote parameters of “true” probability functions, *p*(…), and *φ* to denote parameters of approximations to “true” probability functions, *q*(…). When a probability function directly depends on a parameter, we write the parameter in a subscript before the parentheses; e.g., *p*_*θ*_(…) and *q*_*φ*_(…). We use a line over a value, parameter, or random variable to denote that it is equal to a specific value; e.g., $\bar{z}=1,432$. We use a breve over a parameter or random variable to denote that it should be considered a fixed parameter or random variable within that equation; e.g., $\overset{⌣}{\theta }$ or $\overset{⌣}{z}$. For parameters, this means that they attain a specific value, $\bar{\theta }$; i.e., $\overset{⌣}{\theta }$ means that $\theta =\bar{\theta }$. For random variables, it means that the probability functions with which this variable is associated is considered fixed within a given equation. We use capital letters to denote sets; e.g., $A=\left\{1,\ldots ,\bar{n}\right\}$. We use a superscript with curly brackets to denote indexes; e.g. *z*^{*i*}^ would denote the i’th latent random variable. Similarly, we use a superscript with curly brackets and two numbers separated by a semicolon to denote a set of indexes values; i.e., $z^{\left\{1;\bar{n}\right\}}=z^{\left\{A\right\}}=\left\{z^{\left\{1\right\}},\ldots ,z^{\left\{\bar{n}\right\}}\right\}$. We use a backslash, \, after a set followed by a value, random variable, or parameter to denote the exclusion of that value, random variable, or parameter from that set; i.e., $z^{\left\{A\right\}}/z^{\left\{\bar{n}\right\}}=\left\{z^{\left\{1\right\}},\ldots ,z^{\left\{\bar{n}-1\right\}}\right\}$. We use capital *C* to denote a collection of latent random variables, observed random variables, and parameters. We specify such a collection by enclosing variables and parameters with curly brackets around and with a semicolon separating latent random variables, observed random variables, and parameters in that order; e.g., *C*={*Z*;*X*;Θ}. We use Pa, Ch, An, and De as abbreviations for parent, child, ancestors, and descendants, respectively, and use, e.g., PaΘ(*C*) to denote the set of parameters parent to the collection *C*, and Ch*X*(*Z*) to denote the set of observed variables that are children of the latent random variable *Z*.

### Probabilistic programs

At the heart of our framework, we have chosen to place probabilistic programs. One definition of probabilistic programs is as follows:

*“*probabilistic programs are usual functional or imperative programs with two added constructs: (1) the ability to draw values at random from distributions, and (2) the ability to condition values of variables in a program via observations.”^21^

With these two constructs, any functional or imperative program can be turned into a simultaneous representation of a joint distribution, *p*_Θ_(*Z*,*X*), and conditional distribution, *p*_Θ_(*X*|*Z*), where *X* represent the conditioned/observed random variables, *Z* the unconditioned/latent random variables, and Θ other parameters in the program that are not given a probabilistic treatment, allowing us to integrate classical control constructs familiar to any programmer, such as if/else statements, loops, and recursions into probabilistic models. Such probabilistic programs can express exactly the same functionality as any deterministic programs can and even more. These two constructs are usually provided as extensions to a given programming language through special sample and observe functions or keywords.^19^ Thus, it would be natural to represent such probabilistic programs by pseudo-code. However, based on experience, it can be hard to follow the generative flow of random variables in such pseudo-code. Alternatively, such generative flows have classically been represented by directed graphical models.^22^ Unfortunately, we also found that the semantics of classical directed graphical models do not provide an appropriate presentation.

#### Generative flow graphs

We found that combining the semantics of classical directed graphical models with the semantics of flowcharts into a hybrid representation is a good visual representation. Directed graphical models represent the conditional dependency structure of a model, and flowcharts represent the steps in an algorithm or workflow. The hybrid representation illustrates the order in which samples of random variables in a probabilistic program are generated and how these samples influence the distributions used to generate other samples. For this reason, we call this hybrid representation a generative flow graph.

**Figure 3:**
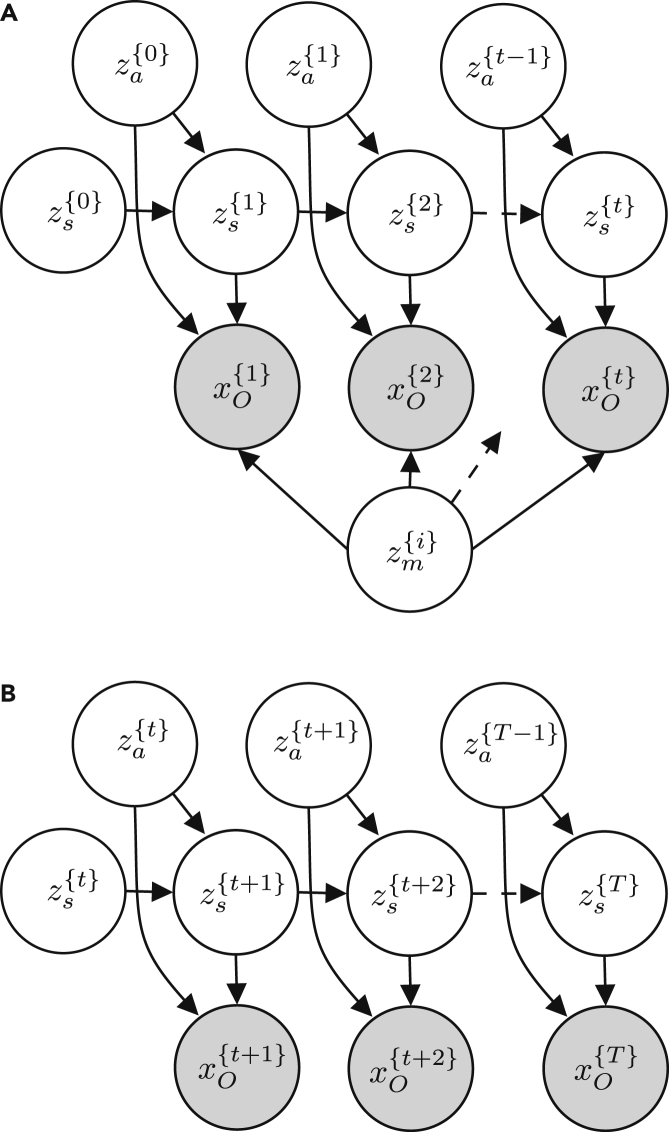
Graphical models with same structure Shown are examples of two directed graphical models developed in different research areas. (A) Graph idiom for the classical simultaneous localization and mapping (SLAM) problem.^23^$z_{s}^{\left\{t\right\}}$ is the state at time *t*, $z_{a}^{\left\{t\right\}}$ is the action at time *t*, $z_{map}^{\left\{i\right\}}$ is the *i*’th pixel in a grid map, and $x_{p}^{\left\{t\right\}}$ is the perceived information at time *t*. (B) Graph idiom for a Markov decision process.^24^$z_{s}^{\left\{t\right\}}$ is the state at time *t*, $z_{a}^{\left\{t\right\}}$ is the action at time *t*, and $x_{O}^{\left\{t\right\}}$ is an “observed” optimality variable at time *t*.

**Figure 4:**
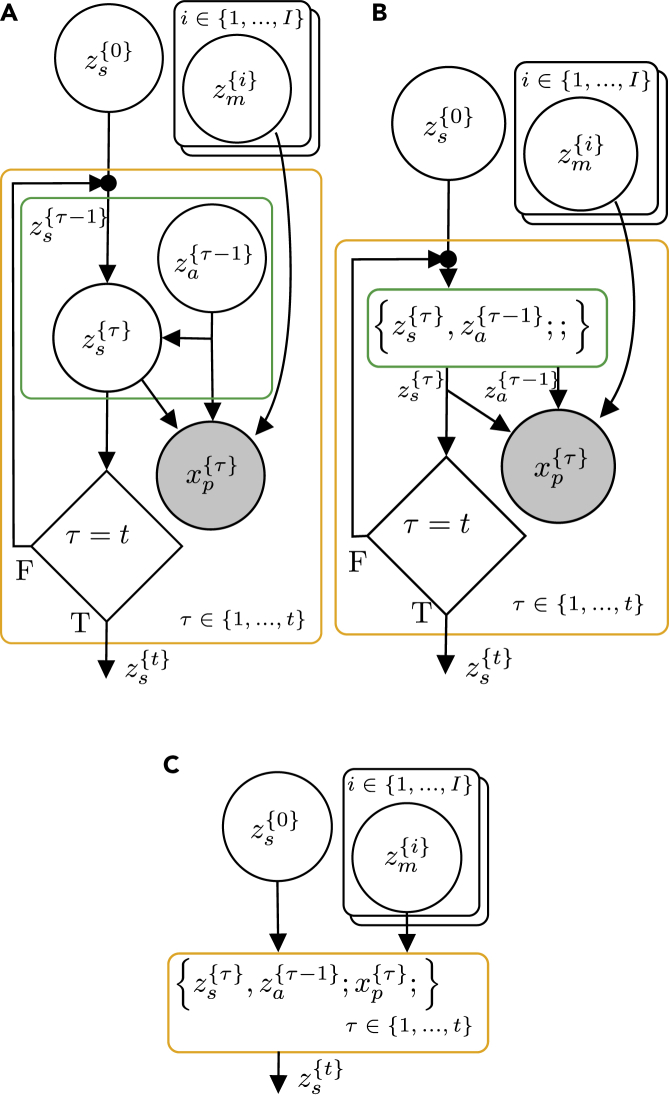
Generative flow graphs for SLAM (A–C) Three semantically equivalent generative flow graphs with different levels of abstractions corresponding to the directed graphical model in Figure 3A.

To exemplify the utility of the generative flow graph representation, consider the graphical model for a classic Markov decision process and the simultaneous localization and mapping (SLAM) problem depicted in Figure 3. With the classic semantics of directed graphical models, it is often the case that size limitations of figures coerce authors to remove some variables from the figure and represent them indirectly by, e.g., dashed arrows, as in Figures 3A and 3B. Similarly, the classic semantics of directed graphical models do not represent the influence from other parameters or variables that are not given a probabilistic treatment even though such variables and parameters might have equal importance for a model. This is especially true when they are not fixed and have to be learned; e.g., when one wants to incorporate artificial neural networks into a model. The classic semantics of directed graphical models also cannot represent dependency structures depending on conditionals giving the illusion that a variable always depends on all of its possible parents and that all variables in the graph are relevant in all situations. Although the semantics of directed graphical models allows us to represent the structure of the joint distribution, *p*(*Z*,*X*), its ability to explicitly express the structure of the posterior distribution, *p*(*Z*|*X*), is limited. Finally, there is no standardized way of representing a fragment of a graphical model, which hinders discussions at different levels of abstraction. Probabilistic programs allow us to easily incorporate the above in our models, and, thus, a more appropriate representation is needed. The semantics of generative flow graphs shown in Table S1 alleviate these problems. Utilizing these semantics, we can redraw the directed graphical model in Figure 3A in multiple ways with different levels of information, as in Figure 4. Note that the choice of node collections is not unique.

One advantage of the semantics of directed graphical models is that, for graphs with no cycles,^22^ such models represent a specific factorization of the joint probability of all of the random variables in the model of the form:

(Equation 1) $$p\left(x^{\left\{1;\bar{n}\right\}},z^{\left\{1;\bar{m}\right\}}\right)=\prod_{n=1}^{\bar{n}}p\left(x^{\left\{n\right\}}|\text{Pa}Z\left(x^{\left\{n\right\}}\right)\right)\prod_{m=1}^{\bar{m}}p\left(z^{\left\{m\right\}}|PaZ\left(z^{\left\{m\right\}}\right)\right)$$

where *x*^{*n*}^ and *z*^{*m*}^ are the n’th observed and the m’th latent random variable in the model, respectively. In principle, this is also true for the generative flow graph representation when it does not contain any cycles, just with the additional explicit representation of dependency on parameters. For generative flow graphs, we can, similar to Equation (1), write up a factorization by including a factor of the form

$$p_{\text{Pa}\theta \left(z^{\left\{m\right\}}\right)}\left(z^{\left\{m\right\}}|\text{Pa}Z\left(z^{\left\{m\right\}}\right)\right)$$

for each latent random variable node *z*^{*m*}^ in the graph, a factor of the form

$$p_{\text{Pa}\theta \left(x^{\left\{n\right\}}\right)}\left(x^{\left\{n\right\}}|\text{Pa}Z\left(x^{\left\{n\right\}}\right)\right)$$

for each observed random variable node *x*^{*n*}^ in the graph, and finally a factor of the form

(Equation 2) $$p_{\text{Θ},\;\text{PaΘ}\left(C^{\left\{k\right\}}\right)}\left(Z,\;X|\text{Pa}Z\left(C^{\left\{k\right\}}\right)\right)$$

for each node collection *C*^{*k*}^={*Z*;*X*;Θ}. If a parent node of *y* is a node collection {*Z*;*X*;Θ}, then Pa*Z*(*y*)=*Z* and Pa*θ*(*y*)=Θ, unless a subset of the variables or parameters in the node collection is explicitly specified next to the parent link. If the internal structure of a node collection is known from somewhere else, then the factor in Equation (2) can of course be replaced by the corresponding factorization. The catch, however, is that a probabilistic program, and, thus, generative flow graphs, can potentially denote models with an unbounded number of random variables and parameters, making it impossible to explicitly write up the full factorization. On the other hand, this just emphasizes the need for alternative ways of representing probabilistic programs other than pseudo-code.

**Figure 5:**
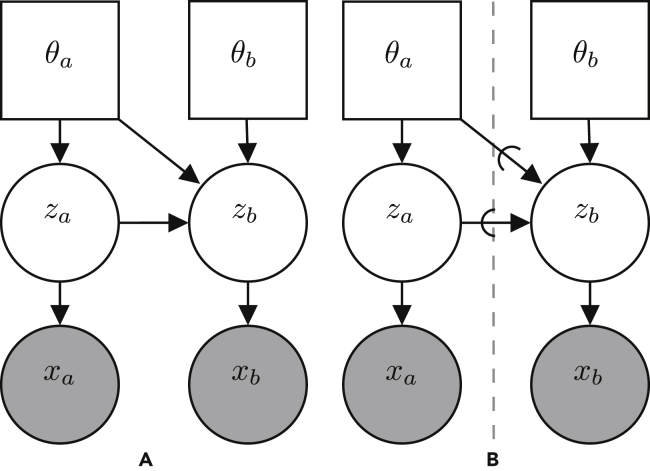
Generative flow graphs with or without a detached link (A and B) Two generative flow graphs representations of a simple model with two parameters, two latent variables, and two observed variables. In both graphs, *θ*_*a*_ and *z*_*a*_ are needed to generate *z*_*b*_, but in (B) we explicitly constrained the inference of *z*_*b*_ to not influence the learning of *θ*_*a*_ and inference of *z*_*a*_. Thus, the evidence provided by *x*_*b*_ is not allowed to have an influence on *θ*_*a*_ and *z*_*a*_. Therefore, the model represented by the nodes on the left side of the dashed line in (B) can be seen as an independent problem.

Besides the possibility of expressing a factorization of the joint prior distribution, the detached link allows us to express additional structure for the posterior distribution *p*(*z*|*x*). Consider the two generative flow graphs in Figure 5. By applying standard manipulations, we can obtain the factorization in Equation (3) for the graph in Figure 5A.

(Equation 3) $$\begin{array}{l} p_{\theta_{a},\theta_{b}}\left(z_{a},\;z_{b}|x_{a},\;x_{b}\right)\\ \;=p_{\theta_{a},\;\theta_{b}}\left(z_{b}|z_{a},\;x_{a},\;x_{b}\right)p_{\theta_{a},\;\theta_{b}}\left(z_{a}|x_{a},\;x_{b}\right)\\ \;=p_{\theta_{a},\;\theta_{b}}\left(z_{b}|z_{a},\;x_{b}\right)p_{\theta_{a},\theta_{b}}\left(z_{a}|x_{a},\;x_{b}\right) \end{array}$$

whereas from the definition of the detached link, we can write the factorization in Equation (4) for the graph in Figure 5B.

(Equation 4) $$\begin{array}{l} p_{\theta_{a},\theta_{b}}\left(z_{a},\;z_{b}|x_{a},\;x_{b}\right)\\ \;=p_{{\overset{˘}{\theta }}_{a},\;\theta_{b}}\left(z_{b}|{\overset{˘}{z}}_{a},\;{\overset{˘}{x}}_{a},\;x_{b}\right)p_{\theta_{a}}\left(z_{a}|x_{a}\right)\\ \;=p_{{\overset{˘}{\theta }}_{a},\;\theta_{b}}\left(z_{b}|{\overset{˘}{z}}_{a},\;x_{b}\right)p_{\theta_{a}}\left(z_{a}|x_{a}\right) \end{array}$$

The main difference between these two factorizations is the distribution over the latent variable *z*_*a*_. In Equation (3), the distribution over the latent variable *z*_*a*_ depends on the evidence provided by observations *x*_*a*_ and *x*_*b*_ and is influenced by parameters *θ*_*a*_ and *θ*_*b*_. In Equation (4), the distribution over *z*_*a*_ depends only on the evidence provided by the observation *x*_*a*_ and is only influenced by the parameter *θ*_*a*_. Therefore, the inference problem of obtaining the posterior distribution over *z*_*a*_ is independent of the inference problem of obtaining the posterior distribution over *z*_*b*_ but not conversely. In general, for a model consisting of $\bar{a}$ node collections, $C^{\left(a\right)}=\left\{Z^{\left(a\right)};\;X^{\left(a\right)};\;{\text{Θ}}^{\left(a\right)}\right\}$, connected only by detached links, we can write the factorization of the posterior as

(Equation 5) $$p_{\text{Θ}}\left(Z|X\right)=\prod_{a=1}^{\bar{a}}p_{{\text{Θ}}^{\left\{a\right\}},\;Pa\overset{˘}{\text{Θ}}\left(C^{\left\{a\right\}}\right)}\left(Z^{\left\{a\right\}}|Pa\overset{˘}{Z}\left(C^{\left\{a\right\}}\right),\;X^{\left\{a\right\}}\right)$$

where the breves are used to emphasize that the variables and parameters are related through a detached link. The possible benefit of being able to express such structure will become clear under Inference algorithms.

Another benefit of the generative flow graph representation is to express models by different levels of abstraction. As an example, consider the three different factorization of the simultaneous localization and mapping problem given in (Equation 6), (Equation 7), (Equation 8):

(Equation 6) $$\begin{array}{l} p\left(z_{s}^{\left\{0;\;t\right\}},\;z_{a}^{\left\{0;\;t-1\right\}},\;x_{p}^{\left\{1;\;t\right\}},\;z_{m}^{\left\{0;\;I\right\}}\right)\\ \;=p\left(z_{s}^{\left\{0;\;t\right\}},\;z_{a}^{\left\{0;\;t-1\right\}},\;x_{p}^{\left\{1;\;t\right\}}|z_{s}^{\left\{0\right\}},\;z_{m}^{\left\{0;I\right\}}\right) \end{array}$$

(Equation 7) $$\cdot p\left(z_{s}^{\left\{0\right\}}\right)\prod_{i=1}^{I}p\left(z_{m}^{\left\{i\right\}}\right)=p\left(z_{s}^{\left\{0\right\}}\right)\prod_{i=1}^{I}p\left(z_{m}^{\left\{i\right\}}\right)$$

(Equation 8) $$\begin{array}{l} \;\cdot \prod_{\tau =1}^{t}\left(\begin{array}{l} p\left(x_{p}^{\left\{\tau \right\}}|z_{s}^{\left\{\tau \right\}},\;z_{a}^{\left\{\tau -1\right\}},\;z_{m}^{\left\{0;\;I\right\}}\right)\\ \;\cdot p\left(z_{s}^{\left\{\tau \right\}},\;z_{a}^{\left\{\tau -1\right\}}|z_{s}^{\left\{\tau -1\right\}}\right) \end{array}\right)\\ \;=p\left(z_{s}^{\left\{0\right\}}\right)\prod_{i=1}^{I}p\left(z_{m}^{\left\{i\right\}}\right) \end{array}$$

$$\;\cdot \overset{t}{\underset{\tau =1}{\prod }}\left(\begin{array}{l} p\left(x_{p}^{\left\{\tau \right\}}|z_{s}^{\left\{\tau \right\}},\;z_{a}^{\left\{\tau -1\right\}},\;z_{m}^{\left\{0;\;I\right\}}\right)\\ p\left(z_{s}^{\left\{\tau \right\}}|z_{a}^{\left\{\tau -1\right\}},\;z_{s}^{\left\{\tau -1\right\}}\right)p\left(z_{a}^{\left\{\tau -1\right\}}\right) \end{array}\right)$$

The generative flow graphs in Figures 4A–4C correspond directly to the factorization in Equations (8), (7), and (6), respectively. They represents different levels of abstractions for the same model. Generative flow graphs simply yield better expressibility compared with their directed graphical model counterparts.

#### Probabilistic programming idioms

We already discussed how probabilistic programming idioms can be seen as a means to achieve functional elegance. In this section, we describe how such idioms can be discovered by inspecting generative flow graphs. We define probabilistic programming idioms as follows:

*“*Probabilistic programming idioms are reusable code fragments of probabilistic programs sharing an equivalent semantic role in their enclosing probabilistic programs.*”*

**Figure 6:**
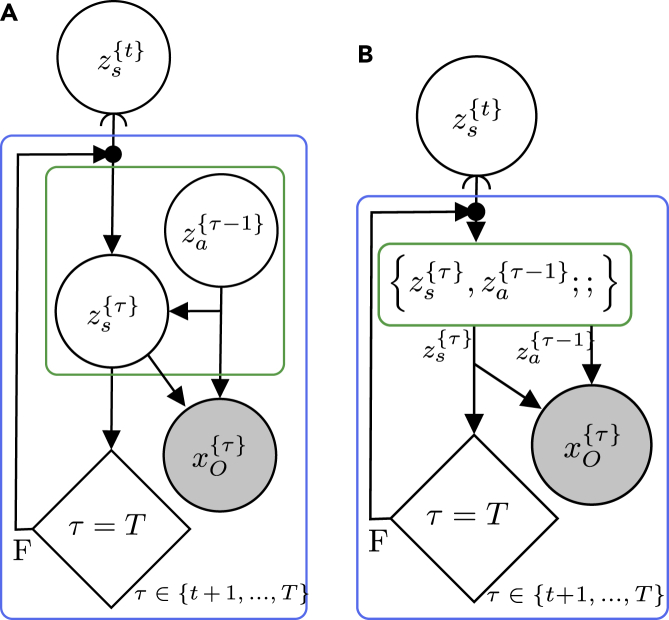
Generative flow graphs for a Markov decision process (A and B) Two semantically equivalent generative flow graphs corresponding to the directed graphical model in Figure 3B.

To identify such probabilistic programming idioms, we can look for node collections containing the same nodes and with the same internal structure in at least two different probabilistic programs. Consider, for example, the node collection $\left\{z_{s}^{\left\{\tau \right\}},\;z_{a}^{\left\{\tau -1\right\}};;\right\}$, highlighted with a green border in the generative flow graph for the SLAM problem and Markov decision process depicted in Figures 4 and 6, respectively. From Figures 4A and 6A, it is clear that the internal structure of this node collection is identical in both graphs and that it represents the factorization

$$p\left(z_{s}^{\left\{\tau \right\}}|z_{s}^{\left\{\tau -1\right\}},\;z_{a}^{\left\{\tau -1\right\}}\right)p\left(z_{a}^{\left\{\tau -1\right\}}\right).$$

Assuming that the distributions $p\left(z_{s}^{\left\{\tau \right\}}|z_{s}^{\left\{\tau -1\right\}},\;z_{a}^{\left\{\tau -1\right\}}\right)$ and $p\left(z_{a}^{\left\{\tau -1\right\}}\right)$ are the same in both models, we could possible create a probabilistic program for this node collection once and then re-use it in both models. This probabilistic program should then take a sample $z_{s}^{\left\{\tau -1\right\}}$ as input. From this input, the program could sample $z_{s}^{\left\{\tau \right\}}$ and $z_{a}^{\left\{\tau -1\right\}}$ from “hard-coded” distributions $p\left(z_{s}^{\left\{\tau \right\}}|z_{s}^{\left\{\tau -1\right\}},\;z_{a}^{\left\{\tau -1\right\}}\right)$ and $p\left(z_{a}^{\left\{\tau -1\right\}}\right)$ using the sample function or keyword of the probabilistic programming language. Finally, the program should return both of these samples. Although this approach might work perfectly for some applications, the two distributions $p\left(z_{s}^{\left\{\tau -1\right\}}|z_{s}^{\left\{\tau -1\right\}},\;z_{a}^{\left\{\tau -1\right\}}\right)$ and $p\left(z_{a}^{\left\{\tau -1\right\}}\right)$ are usually application specific, limiting the usability for an idiom in which they are “hard coded.” A far more general approach would be to allow the probabilistic program to instead take the two distributions as input or having these distributions as free variables, allowing us to re-use the code fragment even for problems where these distributions are not necessarily the same. Rather than fully defining a model of a part of cognition, such a probabilistic program would constitute a template method for the generative flow of that part of cognition. Specific utilization of the model could then be done via a function closure specifying the free distributions. Although the benefits of this example might arguably be limited because the internal structure of the node collection is relatively simple, it is not hard to imagine more complex structures. Consider, for instance, the node collection highlighted with a blue border in Figure 6. By constructing an appropriate probabilistic program for this node collection, we have defined a probabilistic programming idiom constituting the foundation for optimal control and reinforcement learning.

**Figure 7:**
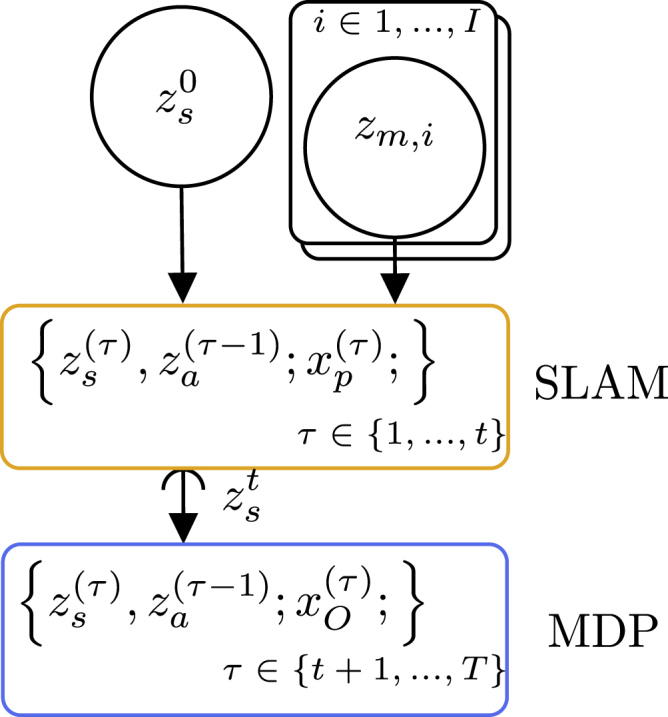
Composition of generative flow graphs A combination of the generative flow graphs for the SLAM problem and the Markov decision process shown in Figures 4 and 6, respectively, could potentially constitute end-to-end navigation behavior for a mobile robot.

When we have developed such probabilistic programming idioms, it empowers us to mix and match them to construct higher-level intelligence without worrying about all details of the underlying models. Figure 7 implies that the output of a specific model for the SLAM problem is used as the input to a Markov decision process but leaves out details about the internal structures.

### Inference algorithms

As stated under Probabilistic programs, a probabilistic program is a simultaneous representation of a joint distribution *p*_Θ_(*Z*,*X*) and a conditional distribution *p*_Θ_(*X*|*Z*). Having defined a model as such distributions, we are usually interested in answering queries about the unconditioned/latent random variables *Z*, given information about the conditioned/observed random variables $X=\bar{X}$. In the combined navigation problem illustrated in Figure 7, we are interested in determining which action to take, $z_{a}^{\left\{\tau \right\}}$, given prior perceived information $x_{p}^{\left\{\tau \right\}}$ for *τ*∈1,…,*t* and future optimality variables $x_{O}^{\left\{\tau \right\}}$ for *τ*∈*t*+1,…,*T*. Often queries of interest are statistics, such as the posterior mean and variance of specific random variables or the posterior probability of a random variable being within a given set. Still, it could also simply be to sample from the posterior, pΘ(Z|X=X¯). All of these queries are somehow related to the posterior distribution given by

(Equation 9) $$p_{\text{Θ}}\left(Z|X=\bar{X}\right)=\frac{p_{\text{Θ}}\left(X=\bar{X},\;Z\right)}{p_{\text{Θ}}\left(X=\bar{X}\right)}=\frac{p_{\text{Θ}}\left(X=\bar{X},\;Z\right)}{\int p_{\text{Θ}}\left(X=\bar{X},Z\right)dZ}.$$

The marginalization by the integral in the denominator of Equation (9) in general does not have an analytical solution or is intractable to computation in most realistic problems, and approximate inference is therefore necessary.^25^ Over time, an abundance of algorithms has been developed to find an approximation to the posterior in specific problems. Unfortunately, many of these algorithms cannot be applied to general probabilistic programs, mainly because of the possible unbounded number of random variables.^19^ Possible applicable inference algorithms can roughly be divided into Monte Carlo-based algorithms, such as sequential Monte Carlo, Metropolis-Hastings, and Hamiltonian Monte Carlo, and optimization-based variational inference algorithms, such as stochastic variational inference. As the size and complexity of models of cognition increase, the computational efficiency of inference algorithms becomes a paramount necessity to achieve sufficient efficiency of the framework presented under Generalized cognitive hourglass model. Although Monte Carlo methods often converge on the true posterior in the limit, convergence can be slow. Conversely, variational inference algorithms are often faster, although they can suffer from simplified posterior approximations.^25^ Also, because variational inference methods are based on optimization, they provide a natural synergy with data-driven discriminative techniques such as deep learning. By accepting that robots are not necessarily supposed to behave optimally but should behave as agents with bounded rationality, this characteristic makes variational inference algorithms an especially interesting choice for cognitive robotics. Variational inference is devoted to giving the reader an introduction to the overall concept of variational inference. Message-passing and Stochastic variational inference present two specific solution approaches commonly used in variational inference: message-passing algorithms and stochastic variational inference, respectively. Both approaches have their weaknesses. Therefore, under Stochastic message-passing, we outline a way of combining these two approaches to overcome their weaknesses. The idea of combining message-passing with stochastic variational inference we have presented before,^26^ but here we generalize the idea to generative flow graphs.

#### Variational inference

Variational inference is an optimization-based approach to approximate one distribution *p*(*Z*) by another simpler distribution *q*(*Z*). *q*(*Z*) is usually called the variational distribution. In general, variational inference is not only used to approximate conditional distribution $p\left(Z|X=\bar{X}\right)$, as in Equation (9). However, with the presented framework in mind, we limit our presentation to this case and focus on variational inference problems in the form

(Equation 10) $$q^{\text{∗}}\left(Z\right)=\text{arg}\underset{q\left(Z\right)\in Q}{\text{min}}D\left(p_{\text{Θ}}\left(Z|X=\bar{X}\right)\|q\left(Z\right)\right)$$

where *D* is a measure of the similarity between *p* and *q* often called a divergence measure, and *Q* is the family of variational distributions from which the approximation should be found. The notation *D*(*p*||*q*) denotes a divergence measure and that the order of the arguments, *p* and *q*, matters. The choice of the family of variational distributions, *Q*, is a compromise between computational efficiency and how precise an approximation one wants. *Q* should be chosen so that we can easily answer given queries. It is important to stress that any variational inference method is more or less biased via the choice of the family of variational distributions *Q*. As a consequence, we cannot view the original model in isolation and have to consider the variational distribution *q*(*Z*) as an implicit part of the cognitive model. Besides the family of variational distributions, the choice of the divergence measure *D* can substantially affect the properties of the approximation. However, empirical results suggest that, for the family of α divergences, subsuming the commonly used Kullback-Leibler divergence, all choices will give similar results as long as the approximating family *Q* is a good fit for the true posterior distribution.^27^

#### Message-passing

Message-passing algorithms solve a possible complicated variational inference problem as defined by Equation (10) by breaking it down into a series of more tractable sub-problems.^27^ The methods are known as message-passing algorithms because of the way the solution to one sub-problem is distributed to the other sub-problems. Message-passing algorithms assume that the model of a problem, *p*(*Z*|*X*), can be factorized into a product of probability distributions

(Equation 11) $$p\left(Z|X\right)=\prod_{a\in A}p^{\left\{a\right\}}\left(Z|X\right).$$

This factorization need not be unique, and each factor *p*^{*a*}^(*Z*|*X*) can depend on any number of variables of *p*(*Z*|*X*). The variational distribution *q*(*Z*) should be chosen so that it factorizes into a similar form

(Equation 12) $$q\left(Z\right)=\prod_{a\in A}q^{\left\{a\right\}}\left(Z\right).$$

With these assumptions, define the product of all other than the *a*’th factor of *q*(*Z*) and *p*(*Z*|*X*), respectively, as

(Equation 13) $$q^{\setminus a}\left(Z\right)=\prod_{b\in A/a}q^{\left\{b\right\}}\left(Z\right),$$

(Equation 14) $$p^{\setminus a}\left(Z|X\right)=\prod_{b\in A\setminus a}p^{\left\{b\right\}}\left(Z|X\right).$$

With these definitions, it is possible to rewrite the problem in Equation (10) into a series of approximate sub-problems on the form.

(Equation 15) $$\begin{array}{l} q^{\left\{a\right\}\text{∗}}\left(Z\right)\approx \\ \;\text{arg}\underset{q^{\left\{a\right\}\in Q^{\left\{a\right\}}}}{\text{min}}D\left[p^{\left\{a\right\}}\left(Z|X\right)q^{\setminus a}\left(Z\right)\|q^{\left\{a\right\}}\left(Z\right)q^{\setminus a}\left(Z\right)\right] \end{array}$$

where *q*^\*a*^(*Z*) is assumed to be a good approximation and, thus, is kept fixed. If the factor families *Q*^{*a*}^ from which *q*^{*a*}^ can be chosen have been chosen sensibly, then the problem in Equation (15) can be more tractable than the original problem, and an approximate solution to the original problem can then be obtain by iterating over these coupled sub-problems, as shown in Algorithm 1. In principle, we can even use different divergence measures for each sub-problem to do mismatched message-passing, which could make some of the sub-problems easier to solve, as described previously.^25^

Algorithm 1 **Pseudo-code for the generic message-passing algorithm. The loop in line 2 can potentially be run in parallel and in a distributed fashion**

1 Initialize *q*^{*a*}∗^(*Z*) for all *a* ∈ *A*.
2 **Repeat**.
3 Pick a factor *A* ∈ *A*.
4 Solve Equation (15) to find *q*^{*a*}∗^(*Z*)
5 **until***q*^{*a*}∗^(*Z*) converges for all *a* ∈ *A*.

In general, the approach is not guaranteed to converge, and Equation (15) might still be a hard problem to solve. In the past, this has limited the approach to relatively simple problems, such as fully discrete or Gaussian problems, for which Equation (15) can be solved analytically.^27^ Therefore, the true power of the method is the principal way in which it allows solving problems in a distributed and parallel fashion, which can be a huge benefit for large models. If the sub-problems are sparsely connected, meaning that sub-problems do not depend on the solution to all of the other sub-problems, then the amount of communication needed can be significantly reduced.

#### Stochastic variational inference

The approach taken by Stochastic Variational inference (SVI) is to reformulate a variational inference problem—e.g., Equations (10) or (15)—to a dual maximization problem with an objective *L* that can be solved with stochastic optimization.^28^ SVI assumes that the variational distribution *q* is parameterized by some parameters Φ. To obtain the dual problem and the objective function *L* of the resulting maximization problem, the steps and assumptions needed depend on whether we have chosen the Kullback-Leibler divergence^28^, ^29^, ^30^ or α divergences.^31^ Regardless, the resulting problem ends up being in the form

(Equation 16) $${\text{Φ}}^{\text{∗}}=\text{arg}\underset{\text{Φ}}{\text{max}}\;\underset{E_{Z∼q_{\text{Φ}}\left(Z\right)}\left[l\left(Z,\;\text{Θ},\;\text{Φ}\right)\right]}{\underset{︸}{L\left(p_{\text{Θ}}\left(Z,\;X=\bar{X}\right),\;q_{\text{Φ}}\left(Z\right)\right)}}.$$

This dual objective function *L* does not depend on the posterior $p_{\text{Θ}}\left(Z|X=\bar{X}\right)$ but only on the variational distribution *q*_Φ_(*Z*) and the unconditional distribution $p_{\text{Θ}}\left(Z,X=\bar{X}\right)$, making the problem much easier to work with. Besides being dual to Equation (10), for the family of α divergences with *α*>0, *L* is also a lower bound on the log evidence $log\left(p_{\text{Θ}}\left(Z\right)\right)$.^31^ Because the log evidence is a measure of how well a model fits the data, we can instead consider the optimization problem^32^

(Equation 17) $${\text{Θ}}^{\text{∗}},\;{\text{Φ}}^{\text{∗}}=\text{arg}\underset{\text{Θ},\;\text{Φ}}{\text{max}}\;\underset{E_{Z∼q_{\text{Φ}}\left(Z\right)}\left[l\left(Z,\text{Θ},\text{Φ}\right)\right]}{\underset{︸}{L\left(p_{\text{Θ}}\left(Z,\;X=\bar{X}\right),\;q_{\text{Φ}}\left(Z\right)\right)}}.$$

which allows us to simultaneously fit the posterior approximation *q*_Φ_ and model parameters Θ to the data $\bar{X}$. An unbiased estimate of the gradient $\bar{\nabla_{W}L}$ of this dual objective *L*, where *W*={Θ,Φ}, can be obtained by utilizing the REINFORCE gradient^33^ or the reparameterization trick.^32^^,^^34^^,^^35^ The objective can then be optimized iteratively by stochastic gradient ascent via the update equation

(Equation 18) $$W^{\left\{l\right\}}=W^{\left\{l-1\right\}}+\rho^{\left\{l-1\right\}}{\bar{\nabla_{W}L}}^{\left\{l\right\}}\left(W^{\left\{l-1\right\}}\right)$$

where superscript {*l*} is used to denote the *l*’th iteration. Stochastic gradient ascent converges to a maximum of the objective function *L* when the sequence of learning rates *ρ*^{*l*−1}^ follow the Robbins-Monro conditions given by

(Equation 19) $$\sum_{l=1}^{\infty }\rho^{\left\{l\right\}}=\infty ,\;\sum_{l=1}^{\infty }{\left(\rho^{\left\{l\right\}}\right)}^{2}<\infty .$$

Because Equation (17) is dual to the original minimization problem, this maximum also provides a solution to the original problem. Although Robbins-Monro conditions are satisfied, it is often necessary to apply variance reduction methods to obtain unbiased gradient estimators with sufficiently low variance. Reduction methods can often be applied automatically by probabilistic programming libraries/languages such as Pyro.^36^ One benefit of solving variational inference problems with stochastic optimization is that noisy gradient estimates are often relatively cheap to compute because of, e.g., subsampling of data. Another benefit is that use of noisy gradient estimates can cause algorithms to escape shallow local optima of complex objective functions.^28^ The downside of SVI is that it is inherently serial and that it requires the parameters to fit in the memory of a single processor.^37^ This could potentially be a problem for cognitive robotics, where large models with lots of variables and parameters are presumably necessary to obtain a high level of intelligence and where queries have to be answered within different time scales; i.e. signals to motors have to be updated frequently, whereas high-level decisions can be allowed to take longer.

#### Stochastic message-passing

To summarize the previous sections, message-passing algorithms exploit the dependency structure of a given variational inference problem to decompose the overall problem into a series of simpler variational inference sub-problems that can be solved in a distributed fashion.^27^ Message-passing algorithms do not give specific directions on how to solve these sub-problems and, thus, classically required tedious analytical derivations that effectively limit the usability of the method. On the other hand, modern SVI methods directly solve such variational inference problems by utilizing stochastic optimization while learning model parameters. By fusion of these two approaches, we could potentially overcome the serial nature of SVI to solve large-scale complex problems in a parallel and distributed fashion. However, to do so, we need to find an appropriate factorization of a given problem. Again, we can make use of the semantics of generative flow graphs. Assuming that we can divide all nodes of a given generative flow graph into a set *C*^{*A*}^ of node collections $C^{\left\{a\right\}}=\left\{Z^{\left\{a\right\}};\;X^{\left\{a\right\}};\;{\text{Θ}}^{\left\{a\right\}}\right\}$ and a set of “global” observed variable nodes *X*_*G*_ having more than one node collection as parent, we can write the posterior factorization

(Equation 20) $$\begin{array}{l} p_{\text{Θ}}\left(Z|X\right)\\ \;=p_{\text{Θ}}\left(Z^{\left\{A\right\}}|X^{\left\{A\right\}},\;X_{G}\right)\\ \;=\frac{1}{p\left(X_{G}|X^{\left\{A\right\}}\right)}p_{\text{Θ}}\left(Z^{\left\{A\right\}},\;X_{G}|X^{\left\{A\right\}}\right)\\ \;=\frac{p\left(X_{G}|Z^{\left\{A\right\}},\;X^{\left\{A\right\}}\right)}{p_{\text{Θ}}\left(X_{G}|X^{\left\{A\right\}}\right)}p_{\text{Θ}}\left(Z^{\left\{A\right\}}|X^{\left\{A\right\}}\right)\\ \;=\frac{p\left(X_{G}|Z^{\left\{A\right\}}\right)}{p_{\text{Θ}}\left(X_{G}|X^{\left\{A\right\}}\right)}p_{\text{Θ}}\left(Z^{\left\{A\right\}}|X^{\left\{A\right\}}\right) \end{array}$$

(Equation 21) $$=\frac{p\left(X_{G}|Z^{\left\{A\right\}}\right)}{p_{\text{Θ}}\left(X_{G}|X^{\left\{A\right\}}\right)}$$

$$\cdot \underset{a\in A}{\prod }p_{{\text{Θ}}^{\left\{a\right\}},\text{Pa}\overset{⌣}{\text{Θ}}\left(C^{\left\{a\right\}}\right)}\left(Z^{\left\{a\right\}}|\text{Pa}\overset{⌣}{Z}\left(C^{\left\{a\right\}}\right),\;X^{\left\{a\right\}}\right)$$

where Equation (20) follows from conditional independence between *X*_*G*_ and *X*^{*A*}^ given *Z*^{*A*}^, and Equation (21) follows from Equation (5). Following the procedure of message-passing, we choose a variational distribution that factorizes as

(Equation 22) $$q_{\text{Φ}}\left(Z\right)=\underset{a\in A}{\prod }q_{{\text{Φ}}^{\left\{a\right\}}}\left(Z^{\left\{a\right\}}\right)$$

In Equation (22), we have exactly one factor for each node collection, and this factor only contains the latent variables of that node collection. This is unlike Equation (12), where a latent variable could be present in multiple factors. By combining Equations (21) and (22), we can write an approximate posterior distribution related to the a’th node collection $p\left(Z|X\right)\approx {\tilde{p}}^{\left\{a\right\}}\left(Z|X\right)$, where

$$\begin{array}{l} {\tilde{p}}_{{\text{Θ}}^{\left\{a\right\}}}^{\left\{a\right\}}\left(Z|X\right)=\\ \;p_{{\text{Θ}}^{\left\{a\right\}},\text{Pa}\overset{⌣}{\text{Θ}}\left(C^{\left\{a\right\}}\right)}\left(Z^{\left\{a\right\}}|\text{Pa}\overset{⌣}{Z}\left(C^{\left\{a\right\}}\right),\;X^{\left\{a\right\}}\right)\\ \;\cdot \frac{p\left(X_{G}|Z^{\left\{A\right\}}\right)}{{\tilde{p}}^{\left\{a\right\}}\left(X_{G}|X^{\left\{a\right\}}\right)}\prod_{b\in A\setminus a}q_{{\overset{˘}{\text{Φ}}}^{\left\{b\right\}}}\left(Z^{\left\{b\right\}}\right) \end{array}$$

${\tilde{p}}^{\left\{a\right\}}\left(X_{G}|X^{\left\{a\right\}}\right)$ is defined in Equation (S1). Based on Equation (15), we can then define approximate sub-problems as

(Equation 23) $$\underset{{\text{Φ}}^{\left\{a\right\}}}{\text{min}}\;D\left[q_{{\text{Φ}}^{\left\{a\right\}}}\left(Z\right)\|{\tilde{p}}_{{\text{Θ}}^{\left\{a\right\}}}^{\left\{a\right\}}\left(Z|X\right)\right]$$

Each of these sub-problems can then be solved successively or in parallel, potentially on distributed compute instances, as outlined in Algorithm 1 and utilizing SVI as described under Stochastic variational inference. To see how this choice of factorization affects the posterior approximations and learning of model parameters Θ, consider the Kullback–Leibler (KL) divergence as a divergence measure. Considering the KL divergence, we can rewrite the objective in Equation (23) as shown in Equation (S2) through Equation (S9) to obtain the following local dual objective for SVI

(Equation 24) $$\begin{array}{l} L_{KL}^{\left\{a\right\}}\left({\text{Θ}}^{\left\{a\right\}},{\text{Φ}}^{\left\{a\right\}}\right)=\\ \;E_{Z∼{\tilde{q}}_{\text{Pa}\overset{˘}{Z}}^{\left\{a\right\}}}\left[LogEvd_{X_{G},\;X^{\left\{a\right\}}}^{\left\{a\right\}}\left({\text{Θ}}^{\left\{a\right\}}\right)\right]-C\\ \;-D_{KL}\left[q_{{\text{Φ}}^{\left\{a\right\}}}\left(Z\right)\|{\tilde{p}}_{{\text{Θ}}^{\left\{a\right\}}}^{\left\{a\right\}}\left(Z|X\right)\right] \end{array}$$

where *C* is a constant with respect to Θ^{*a*}^ and Φ^{*a*}^, and $LogEvd^{\left\{a\right\}}\left(X_{G},\;X^{\left\{a\right\}}\right)$ is the joint log-evidence over global observed variables *X*_*G*_ and observed variables  *X*^{*a*}^ local to the a’th node collection. Because the first term on the right side is constant with respect to Φ^{*a*}^, maximizing this local dual objective with respect to Φ^{*a*}^ will minimize the KL divergence. Because $D_{KL}\left[q_{{\text{Φ}}^{\left\{a\right\}}}\left(Z\right)\|{\tilde{p}}_{{\text{Θ}}^{\left\{a\right\}}}^{\left\{a\right\}}\left(Z|X\right)\right]\geq 0$, by definition, it follows from Equation (24) that

$$\begin{array}{l} E_{Z∼{\tilde{q}}_{\text{Pa}\overset{˘}{Z}}^{\left\{a\right\}}}\left[LogEvd^{\left\{a\right\}}\left(X_{G},\;X^{\left\{a\right\}}\right)\right]-C\\ \;\geq L_{KL}^{\left\{a\right\}}\left({\text{Θ}}^{\left\{a\right\}},{\text{Φ}}^{\left\{a\right\}}\right) \end{array}$$

Therefore, by maximizing the local dual objective $L_{KL}^{\left\{a\right\}}\left({\text{Θ}}^{\left\{a\right\}},{\text{Φ}}^{\left\{a\right\}}\right)$ with respect to the local model parameters Θ^{*a*}^ we push the expected joint log-evidence over the global *X*_*G*_ and the local *X*^{*a*}^ observed variables higher, where the expectation is taken with respect to the joint variational distribution over latent variables parent to the a’th node collection. This means that we can simultaneously fit our local model parameters Θ^{*a*}^ to the evidence and obtain an approximate local posterior distribution $q_{{\text{Φ}}^{\left\{a\right\}}}\left(z^{\left\{a\right\}}\right)$. Although these derivations were made for the KL divergence, similar derivations can be done for the more general family of α divergences.

To evaluate this local dual objective, we only need information related to the local node collection, its parents, and other node collections having the same global observed variables as children, providing substantially computational speedups for generative flow graphs with sparsely connected node collections and global observed variables. To use this procedure with a standard probabilistic programming language, we would have to create a probabilistic program fragment for each node collection, the corresponding variational distribution, and the global observed variables. These fragments would then have to be composed together to form the local objectives, potentially in an automated fashion.

So far, in this section, we assumed that all sub-problems are solved through a variational problem as in Equation (23). However, there are, in principle, no reasons why we could not use estimates of sub-posteriors, *q*(*z*^{*b*}^), obtained through other means in Equation (23), as long as we can sample from these sub-posteriors, making the outlined method very flexible to combine with other methods, but analysis of the results obtained through the combined inference becomes more difficult. It is also important to stress that the factorization used above is not unique. It would be interesting to investigate whether other factorizations could be employed and for which problems these factorizations could be useful.

If we can divide a generative flow graph representing an overall model of cognition into node collections and global observed variables, then we can utilize the combination of message-passing and SVI presented in this section to distribute the computational burden of performing inference within this model. At the same time, we can learn local model parameters, yielding a very flexible tool allowing us to fully specify the part of a model we are certain about and potentially learn the rest.

### Probabilistic programming languages

So far, our focus has been on representation of models defined by probabilistic programs and on how to answer queries related to these models via modern probabilistic inference. However, we have not considered how this is made possible by probabilistic programming languages and their relation to deterministic programming languages. Here we will not give a detailed introduction to probabilistic programming and refer interested readers to other sources.^19^^,^^38^^,^^39^ Instead, we will give a short overview of languages relevant to modeling cognition.

As already mentioned under Probabilistic programs, the main characteristics of a probabilistic program are a construct for sampling randomly from distributions and another construct for condition values of variables in the program. The purpose of probabilistic programming languages is to provide these two constructs and to handle the underlying machinery for implementing inference algorithms and performing inference from these constructs. As with any other programming language, design decisions are not universally applicable or desirable, and different trade-offs are made purposefully to achieve different goals. This fact, combined with theoretical advancements, has resulted in several different probabilistic programming languages. For an extensive list, see van de Meent et al.^19^ Some of these are domain specific, aimed at performing inference in a restricted class of probabilistic programs, such as STAN.^40^ These restrictions are usually employed to obtain more efficient inference. More interesting for the framework presented under Generalized cognitive hourglass model, however, are languages self-identifying as universal or general purpose, such as Pyro^36^ and Venture.^41^^,^^42^ These languages aim to perform inference in arbitrary probabilistic programs, maximizing flexibility for modeling cognition.

A recent trend has been to build probabilistic programming languages on top of deep-learning libraries such as PyTorch^43^ and TensorFlow.^44^ This is done to use the efficient tensor math, automatic differentiation, and hardware acceleration these libraries provide and to get tighter integration of deep-learning models within probabilistic models. Examples of such languages are Pyro^36^ and ProbTorch,^45^ built on PyTorch, and Edward,^46^ built on TensorFlow. Again, when considering use within the framework presented under Generalized cognitive hourglass model, languages based on PyTorch or TensorFlow 2.0 could potentially have an advantage over others because of the dynamic approach to constructing computation graphs. This is because dynamic computation graphs more easily allow us to define dynamic models that include recursion and unbounded numbers of random choices.^19^ Constructs potentially being indispensable for models of higher-level cognition supposed to evolve.

Python, as a high-level general-purpose programming language, makes modeling effortless in these languages. However, being based on Python, the computational efficiency of these languages is potentially limited by the need for interpretation. For this reason, the relatively recent project called NumPyro^47^ is in active development. NumPyro provides a backend to Pyro based on NumPy^48^ and JAX,^49^ which enables just-in-time compilation and, thus, could potentially provide much better computational efficiency, which is essential for any practical robotic system.

The choice of which probabilistic programming language to use depends on the flexibility needed to model cognition. However, universal or general-purpose languages based on deep-learning libraries, possibly with just-in-time compilation and hardware acceleration, seem promising for general modeling of cognition and especially for cognitive robotics.

### Application examples

**Figure 8:**
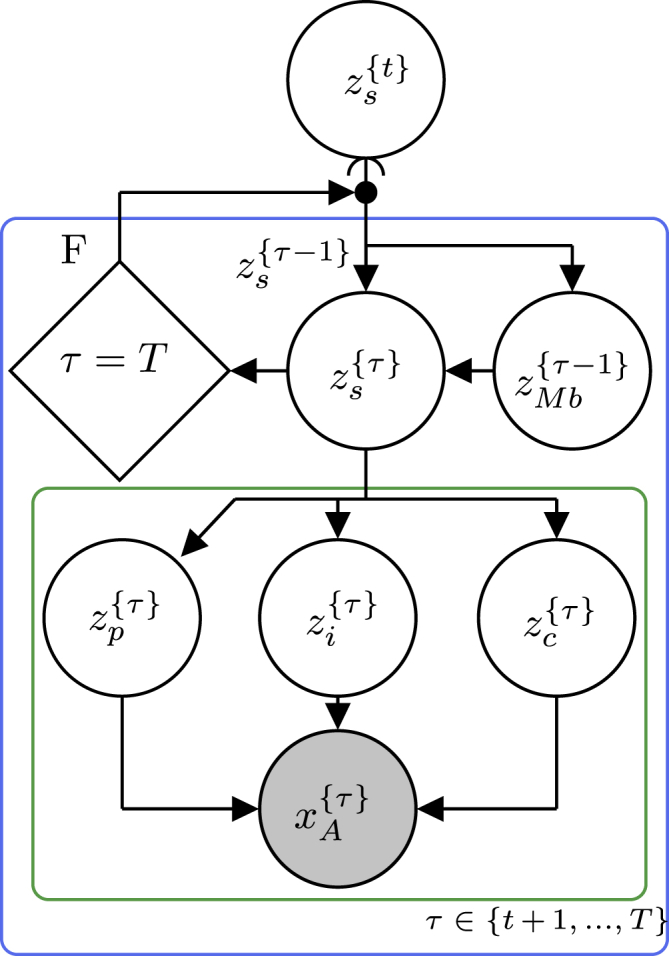
Generative flow graph for planning Shown is an excerpt of the generative flow graph representation of the idiom used in the abstract class “Planning.”^50^ Red, green, and blue relate node collections to the methods of the UML class diagram in Figure 9. The variables $z_{p}^{\left\{\tau \right\}}$, $z_{i}^{\left\{\tau \right\}}$, and $z_{c}^{\left\{\tau \right\}}$ represent progress, information gain, and constraints, respectively. $z_{s}^{\left\{\tau \right\}}$ represents the robot’s internal state representation at time τ. $z_{Mb}^{\left\{\tau \right\}}$ represents the actions of the robot at time τ contained in the “motor buffer” (Mb). $x_{A}^{\left\{\tau \right\}}$ quantifies the amount of attention the robot should give to a given state $z_{s}^{\left\{\tau \right\}}$ through weighting of the progress, information gain, and constraint variables.

**Figure 9:**
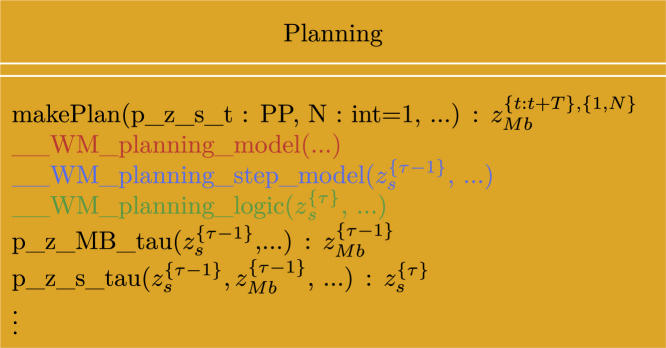
UML class diagram for “planning” Shown is an excerpt of the UML class diagram for the abstract class “Planning.”^50^ For simplicity, we only included the methods relevant for discussion in this manuscript. Italic text designates abstract classes and methods, + designates public methods, PP designates an argument of the type probabilistic program, and red, green, and blue designate the methods implementing the node collections with corresponding colors in Figure 8.

To demonstrate the concepts presented in this paper and the utility of the framework, we have begun an initiative to implement some generally applicable probabilistic programming idioms with basis in the “Standard Model of the Mind,”^10^ which is available as a GitHub repository.^50^ The repository currently contains one such idiom, called “__WM_planning_model(…)” implemented within the “Planning” class. The purpose of this idiom is to provide basic functionality to plan future actions of a robot based on cognitive concepts of desirability, progress, information gain, and constraints. Figure 8 illustrates an excerpt of the generative flow graph representation of the idiom. For an in-depth presentation of the inner workings of the idiom, we refer the reader to our other paper.^51^ As shown in Figure 8, the idiom can be divided into a hierarchical structure of node collections in which the red node collection internally depends on the blue node collection, which, in turn, depends on the green node collection and recursively on itself. Rather than implementing the idiom as one large probabilistic program, this hierarchical structure allows us to implement the idiom as multiple smaller probabilistic programs. To keep the idiom generally applicable, it is implemented within an abstract Python class with a method for each of the node collections shown in Figure 8, which depends on some abstract methods that need to be specified on a per-application basis. Figure 9 shows a simplified UML class diagram of the main methods of the class. The method for each of the node collections in the idiom contains the main structure and functionality of the idiom. However, without implementation of the abstract methods, it is inoperative, and it is implementation of, e.g., the probabilistic program for the state transition “p_z_s_tau(…)” that makes it application specific.

In the simplest use case, the user can use the idiom simply by creating a child class that inherits the “Planning” class and implements the abstract methods. The user can then call the public method “makePlan(…)”, which performs SVI on the idiom and returns K samples from the approximate posterior

(Equation 25) $$z_{Mb}^{\left\{t:t+T\right\},\left\{k\right\}}∼q\left(z_{Mb}^{\left\{t:t+T\right\}}\right)\approx p\left(z_{Mb}^{\left\{t:t+T\right\}}|x_{A}^{\left\{t:t+T\right\}}\right)$$

constituting an optimal plan of future actions according to the idioms notion of progress, information gain, and constraints. The abstract methods that need to be implemented are rather non-restrictive, and most are only assumed to be probabilistic programs developed in Pyro,^36^ making the idiom very versatile. Besides the “Planning” class containing the idiom, the repository also contains applications examples for robot exploration and multi-robot navigation, demonstrating different use cases of the idiom.

#### Robot exploration

**Figure 10:**
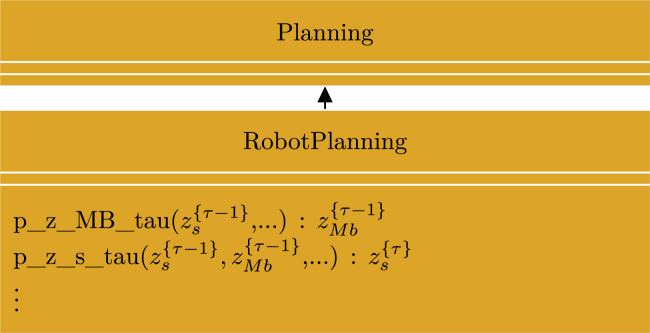
UML class diagram for “RobotPlanning” Shown is an excerpt of the UML class diagram for the class “ RobotPlanning” used for robot exploration.^51^ The model for robot exploration aligned well with the idiom in Figure 8 and could be implemented as a child class inheriting from the abstract “Planning” class.

The purpose of this use case is to demonstrate high-level robot motion planning with the goal of exploring an environment represented by a grid map in the long-term memory with a lidar mounted on a robot.^51^ In this particular case, the model of cognition aligned perfectly with the “__WM_planning_model(…)” idiom. Thus, the application was implemented simply as a child class implementing the abstract methods inherited from the abstract parent class “Planning” as illustrated in Figure 10. Therefore, the implementation for this application was greatly simplified.

**Figure 11:**
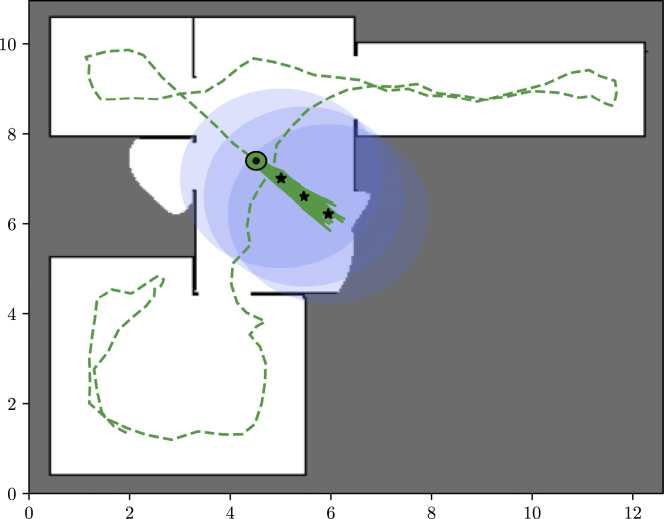
Robot exploration simulation Shown are results of a simulation of high-level robot motion planning with the goal of exploring an unknown environment with a lidar as the perceptual input. Gray indicates unexplored parts of the environment, white indicates unoccupied areas, black indicates obstacles, the green circle with a black border shows the current location of the robot, the green dashed line shows the robot’s past path, the solid green lines indicate samples from the future optimal path distribution, black stars indicate the mean of these samples, and transparent blue circles illustrate the lidar’s range at these positions.

In the related paper,^51^ the approach was tested on 35,126 2D floor plans available in the HouseExpo dataset, utilizing a modified version of the accompanying PseudoSLAM simulator.^52^ Figure 11 shows a snapshot of one of the simulations.

In this extensive simulation study, it was demonstrated that the method was indeed capable of planning actions to guide a robot toward new knowledge, exploring a large part of most of the floor plans. During these simulations, only 0.25‰ of actions taken based on the “__WM_planning_model(…)” idiom resulted in collisions, demonstrating the ability of the approach to avoid constraints. Currently, the implementation for this application uses down to approximately 1 s on planning depending on the settings. This is deemed sufficient for high-level planning in robotics applications, and, thus, this simulation study also hints toward sufficient efficiency of the framework, which will only be corroborated by further code optimization.

#### Multi-robot navigation

**Figure 12:**
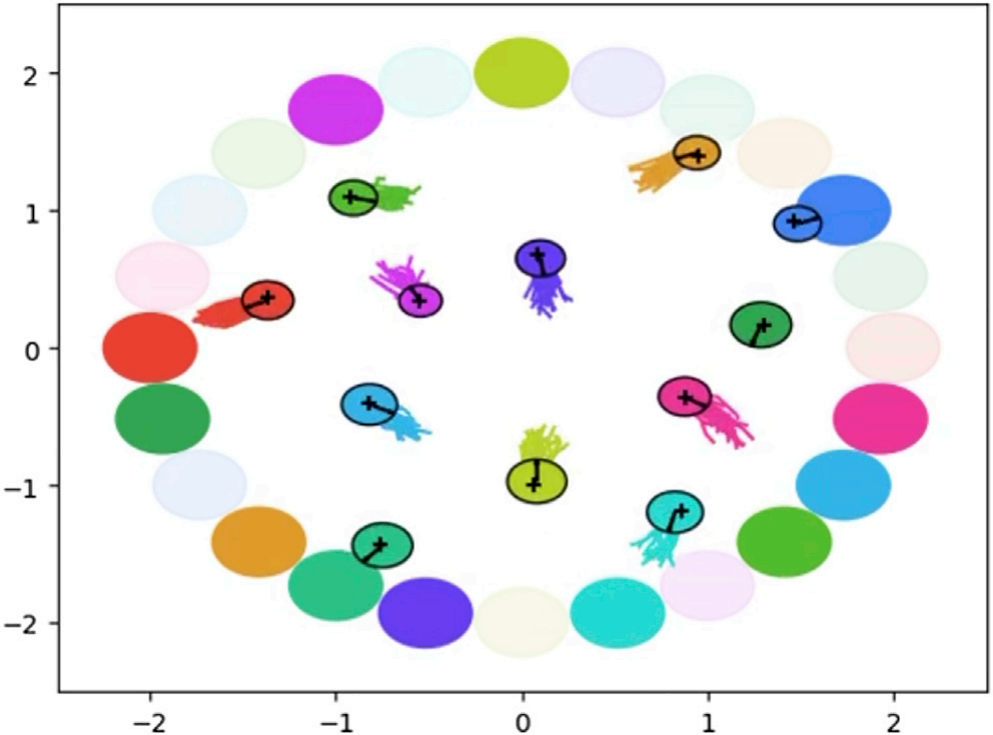
Multi-robot navigation simulation Shown is a snapshot of a simulation with 12 robots utilizing the “Planning” idiom to plan actions toward their goal while avoiding collision with each other. Colored circles with a black border indicate the current location of the robots, solid colored lines indicate samples of their future planned path distribution, colored circles indicate their current goals, and transparent colored circles indicates their last goal.

**Figure 13:**
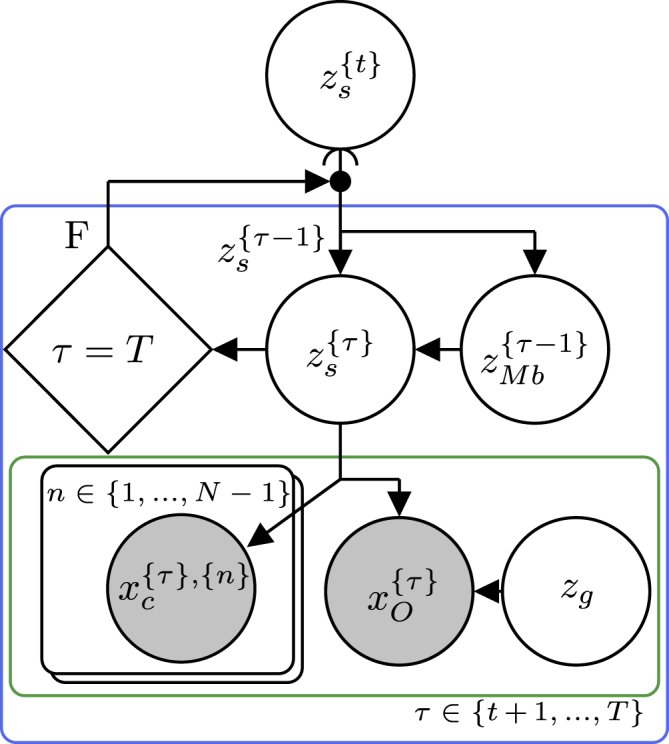
Generative flow graph for multi-robot navigation Shown is a generative flow graph of the model derived for each of the robots in a multi-robot navigation problem.^26^$z_{s}^{\left\{\tau \right\}}$ represents the robots internal representation of its own state as well as the state of the other robots at time τ. $z_{Mb}^{\left\{\tau \right\}}$ represents the actions of the robot itself as well as the communicated planned actions of the other robots at time τ. $x_{O}^{\left\{\tau \right\}}$ quantifies how “optimal” the robot’s own state is in regard to getting closer to its own goal state $z_{g}$. $x_{c}^{\left\{\tau \right\},\left\{n\right\}}$ represents the global constraints of avoiding collision with each of the *N*-1 other robots; i.e. *X*_*G*_ from Stochastic message-passing.

The second application example relies heavily on the stochastic message-passing approach described under Stochastic message-passing to implement a simplistic form of communication between robots.^26^ In this application, N unicycle type robots have to plan low-level actions toward their goals while avoiding collisions with the other robots, given knowledge about the other robots’ expected future path, as illustrated in Figure 12. Figure 13 shows a generative flow graph of the model derived for this problem.^26^

**Figure 14:**
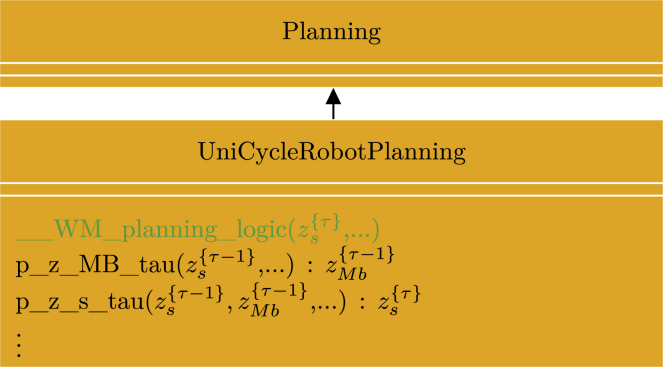
UML class diagram for “UniCycleRobotPlanning” Shown is an excerpt of the UML class diagram for the class “UniCycleRobotPlanning” used for multi-robot navigation.^26^ A large part of the model for multi-robot navigation aligned with the idiom in Figure 8 and could be implemented as a child class inheriting from the abstract “Planning” class overwriting the parts of the model that did not align.

By comparing Figures 13 and 8, it is clear that the models are not exactly the same. However, the differences are encapsulated within the node collections marked by a green boarder in both diagrams. Thus, by creating a child class inheriting the “Planning” class but overwriting the “__WM_planning_logic(…)” method, it was possible to re-use a large part of the “__WM_planning_model(…)” idiom, greatly simplifying the implementation process. Figure 14 illustrates an excerpt of the UML class diagram used for this application. Because the robots in this application had to plan low-level actions and keep track of the state of the other robots, the implementations of abstract methods like, e.g., “p_z_MB_tau(…)” and “p_z_s_tau(…),” also had to be different from the ones used in the robot exploration application.

In the paper related to this use case,^26^ the approach used was verified through an extensive simulation study and a real-world experiment. From simulations of 2–32 robots, it was concluded that the approach performs as well as, if not better than, the state-of-the-art algorithm B-UAVC^53^ made exclusively for the problem of multi-robot collision avoidance. This was despite the fact that the approach required far less analytical analysis because only a relatively simple model of the problem had to be derived before the general concepts for performing inference in such a model presented within this paper could be applied. The approach was also tested in a real-world experiment with two TurtleBot3 Burger robots equipped with an Intel NUC10FNK each for performing the necessary computations. The success of this real-world experiment demonstrated that sufficient computational efficiency is possible on standard hardware as well as the real-world applicability of concepts presented n this paper.

#### Application discussion

The point of these examples is not that the method necessarily performs better than any other method or that the applications could not have been implemented in another way. The point is that, by following the concepts of the framework presented in this manuscript, it is possible to develop generally applicable models of cognition that can easily be adapted and/or extended to new use cases, mitigating the complexity and burden of creating cognitive architectures for robotics applications.

Although the repository currently does not contain a broad range of cognitive capabilities, the two examples demonstrates most of the concepts presented under Probabilistic programs through probabilistic programming languages. Specifically, the examples demonstrate combined usage of probabilistic programming, inference in probabilistic programs, generative flow graphs, probabilistic programming idioms, and stochastic message-passing for two real-world robotics applications.

Besides the present features of the idiom and “Planning” class, based on experiences from solving the multi-robot navigation problem, the “__WM_planning_model(…)” idiom is currently being extended with a desirability variable for reaching goal states and detection of impasse. Currently, the “Planning” class makes use of SVI. However, if, in the future, we want to use another algorithm for inference, we can simply inherit the “Planning” class and overwrite the “makePlan(…)” method to accommodate this inference algorithm. Because the idiom is implemented via the probabilistic programming language Pyro, we do not need to re-implement the idiom itself to accommodate this inference algorithm. All of this, together with the fact that the two vastly different applications are implemented from the same probabilistic programming idiom, demonstrates how models developed in the proposed framework can encourage cooperation and re-use of existing results and inspire new work.

## Discussion

### Conclusion

Inspired by Sigma’s cognitive hourglass model,^4^ we have outlined a framework for developing cognitive architectures for cognitive robotics. With probabilistic programs at the center, this framework is sufficiently general to span the full spectrum of emergent, symbolic, and hybrid architectures. By dividing cognitive architectures into a series of layers, this framework provides levels of abstractions between models of cognition and the algorithms that implement them on computational devices. Some of these layers also directly relate to other fields of research, encouraging better cooperation.

We also presented a graphical representation of probabilistic programs we call generative flow graphs. We showed how such generative flow graphs can help identify important universal fragments of probabilistic programs and models, fragments that could potentially be re-used in development of other cognitive architectures, encouraging cooperation and easier re-use of existing results.

We introduced the problem of inference within probabilistic programs. We briefly reviewed possible approaches and argued that variational inference approaches seem interesting for cognitive robotics. We introduced two commonly used approaches: message-passing and SVI. We also outlined the weaknesses of each approach and proposed a combined approach we call stochastic message-passing. The proposed approach provides a principal way of distributing the computational burden of inference and parameter learning.

To support implementation within the framework, we reviewed existing probabilistic programming languages providing the necessary machinery to implement inference algorithms for and perform inference in probabilistic programs.

Finally, we provided a brief introduction to an initiative that provides evidence of the applicability of the framework and concepts presented within this paper but also functions as a starting point and tool for researchers who want to work within the framework.

The main topics in this paper have been the framework itself, representation of cognitive models, and computational burden. These topics are essential ingredients of the framework and are interesting research directions.

### Limitations of the study

The section Application examples is limited to action selection through planning and control, and to fully demonstrate the flexibility of the proposed framework, applications to other cognitive tasks remain to be demonstrated. We see no reason why the same principles could not be applied to other aspects of cognition, such as perception, attention, memory, social interaction, metacognition, and even emotion.

## Experimental procedures

### Resource availability

#### Lead contact

Requests for further information can be directed to the lead contact, M.R.D., at mrd@es.aau.dk.

#### Materials availability

This study did not generate new unique materials.

## Data Availability

This study did not generate new data or code. However, the data and code related to the two studies presented and discussed under Application examples are available online as a GitHub repository.^50^ More specifically, the data and code related to the robot exploration study presented under Robot exploration are available via Zenodo.^54^ Similarly, the data and code related to the multi-robot exploration study presented under Multi-robot navigation are available at the repository branch.^55^

## Supplemental information

Document S1. Supplemental Experimental Procedures and Table S1

Document S2. Article plus Supplemental information
