# Supplementary material for: Toward an idiomatic framework for cognitive robotics
Source: Patterns (N Y). 2022 Jul 8;3(7):100533. doi: 10.1016/j.patter.2022.100533 (PMC9278519; doi:10.1016/j.patter.2022.100533)
Supplement: Document S1. Supplemental Experimental Procedures and Table S1 [file mmc1.pdf]

Patterns, Volume *3*

Supplemental information

Toward an idiomatic framework for cognitive robotics

Malte Rørmose Damgaard, Rasmus Pedersen, and Thomas Bak

S1. Supplemental Experimental Procedures

S1.1. Generative Flow Graphs

Symbol	Description	Meaning
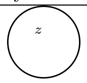	Circle without colored background	A node symbolizing a probabilistic variable, corresponding to a "*sample*" function or keyword in the probabilistic program. We denote this as a "*Latent Variable Node*".
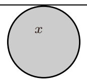	Circle with colored background	A node symbolizing an observed probabilistic variable, corresponding to a "*observe*" function or keyword in the probabilistic program. We denote this as an "*Observed Variable Node*".
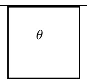	Square without colored background	A node symbolizing learn-able parameters in the probabilistic program. That is model parameters that can change at run-time. We denote this as a "*Variable Parameter Node*"
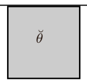	Square with colored background	A node symbolizing fixed parameters in the probabilistic program. Use-full for representing parameters that cannot change at run-time such as tuning parameters. We denote this as a "*Fixed parameter node*"
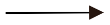	simple arrow	Link showing the generative path in a Probabilistic Program. The arrow can start in Latent Variable Nodes and Parameter nodes, and only point towards *latent variable nodes* and *observed variable nodes*. In large graphs or in cases where the origin of a link can be uncertain the readability can be improved by adding the name of the node from which the link originates next to the link. We denote this as a "*Generative Link*"
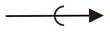	Half circle on arrow	Symbolizing that operations downstream of this link in the probabilistic program should not influence nodes upstream to this link in the generative path. I.e. information such as accumulated gradients in a backward pass of automatic differentiation should not be propagated back through this link, corresponding to a ".detach()" and ".stop_gradient" call in PyTorch and TensorFlow, respectively. We denote this as a "*Detached Link*". A "variable parameter node" having only a detached link, can be considered a "fixed parameter node" or vice versa.
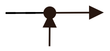	Simple arrow pointing towards another simple arrow	Depending on the context, samples from different *latent variable nodes* may be used to generate the next sample. The generative link from the most recent sampled *latent variable node* in the generative path is the new active link. Used, e.g., to represent for loops.
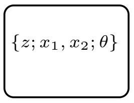	Polygon with rounded corners	Collection of nodes with internal dependency structure. The full structure of links between nodes can be shown inside the polygon. Alternatively, the names of the nodes can be written between curly brackets, $\{z; x; \theta\}$, with a semi-colon separating variables nodes, z, observed nodes, x, and parameter nodes, θ, in that order. Finally, a node collection can also simply be defined somewhere else, $C = \{z; x; \theta\}$, and referred to by, e.g., a single letter, in which case we encourage the use of capital letters to emphasize the difference from the other types of nodes. In cases where one or more types of nodes are not present in a collection, both semi-colons should still be there. E.g., $\{; x_1, x_2;\}$ for a collection the two observed nodes x_1 and x_2. Such a collection of nodes directly corresponds to the factor $p_\theta(x_1, x_2\|z)$ in a factorization of the joint distribution over all variables in a model. We denote this as a "*Node Collection*". A node is only allowed to be within one node collection unless it is within a node collection fully nested within another.
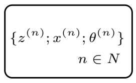	Index specification in one corner of a polygon with rounded corners	Collection of nodes with indexed names. When the index is used to name nodes it is good practice to use the index in a superscript and encapsulate it in round brackets to emphasize that it is an index. We denote such a node collection an "*Indexed Node Collection*". The valid index should be clear from the context. E.g., in the case of a loop around the indexed collection, the index is incremented each time the loop enters the indexed node collection. When no such loops exist around the indexed node collection that could potentially cause ambiguity, it can be used instead of writing all the variables in a node collection with the curly brackets. For an example see Fig. 4c
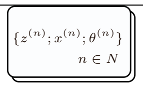	Stacked polygons with rounded corners	Explicitly representation of multiple identical collections of nodes conditionally independent given their parents or simply independent if there are no parents. Here the one index is used for each of the independent collections.
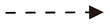	Dashed arrow	A link symbolizing an indirect relation between nodes. Such a link is non-generative, meaning that it is not directly used as a parameter in the generation of other samples, but only influences the generative path of other samples. We denote this as an "*Influence Link*"
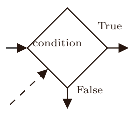	A Polygon with generative links connected at vertices, influence links towards the polygon connected at edges, and conditional values nearby generative links pointing away from the polygon	Node representing a condition changing the "direction" of the generative flow in a probabilistic program. We denote this as "*conditioned generative branching*".
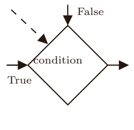	A Polygon with generative links connected at vertices, influence links towards the polygon connected at edges, and conditional values nearby generative links pointing towards the polygon	Node representing a condition selecting one out of two or more possible generative flows from parent nodes. We denote this as "*conditioned generative selection*".

Table S1: Semantics of the Generative Flow Graph representation of probabilistic programs.

S1.2. Rewriting KL-divergence for Stochastic Message-Passing

In this section, we will derive the dual objective for the combination of message-passing and stochastic variational inference presented in Section 6.4. Start by considering

$$
\begin{aligned}
p_\Theta(X_G|X^{\{A\}}) &= \int p_\Theta(X_G, Z^{\{A\}}|X^{\{A\}})dZ \\
&= \int p(X_G|Z^{\{A\}}, X^{\{A\}})p_\Theta(Z^{\{A\}}|X^{\{A\}})dZ \\
&= \int p(X_G|Z^{\{A\}})p_\Theta(Z^{\{A\}}|X^{\{A\}})dZ \\
&= \int p(X_G|Z^{\{A\}}) \prod_{a\in A} p_{\Theta^{\{a\}},\mathrm{Pa}\breve{\Theta}(C^{\{a\}})}\left(Z^{\{a\}}|\mathrm{Pa}\breve{Z}\left(C^{\{a\}}\right), X^{\{a\}}\right) dZ
\end{aligned}
$$

By replacing $p_{\Theta^{\{b\}},\mathrm{Pa}\breve{\Theta}(C^{\{b\}})}\left(Z^{\{b\}}|\mathrm{Pa}\breve{Z}\left(C^{\{b\}}\right), X^{\{b\}}\right)$ with their corresponding variational distributions $q_{\breve{\Phi}^{\{b\}}}\left(Z^{\{b\}}\right)$ for $b \in A \setminus a$ we obtain

$$
\tilde{p}^{\{a\}}(X_G|X^{\{a\}}) = \int p(X_G|Z^{\{A\}})p_{\Theta^{\{a\}},\mathrm{Pa}\breve{\Theta}(C^{\{a\}})}\left(Z^{\{a\}}|\mathrm{Pa}\breve{Z}\left(C^{\{a\}}\right), X^{\{a\}}\right) \prod_{b\in A\setminus a} q_{\breve{\Phi}^{\{b\}}}\left(Z^{\{b\}}\right) dZ \tag{S1}
$$

where we have used $\tilde{p}^{\{a\}}(X_G|X^{\{a\}})$ instead of $\tilde{p}^{\{a\}}(X_G|X^{\{A\}})$ to emphasize the conditional independence between X_G and $X^{\{b\}}$ given $Z^{\{b\}}$ for $b \in A \setminus a$ implicitly assumed by the approximation. Furthermore, also notice that we can rewrite the distribution, $p(X_G|Z^{\{A\}})$, as follows

$$
\begin{aligned}
p(X_G|Z^{\{A\}}) &= p(\mathrm{Ch}X_G\left(Z^{\{a\}}\right)|Z^{\{A\}})p(X_G \setminus \mathrm{Ch}X_G\left(Z^{\{a\}}\right)|Z^{\{A\}}) \\
&= p\left(\mathrm{Ch}X_G\left(Z^{\{a\}}\right)|\mathrm{Pa}Z\left(\mathrm{Ch}X_G\left(Z^{\{a\}}\right)\right)\right) p\left(X_G \setminus \mathrm{Ch}X_G\left(Z^{\{a\}}\right)|Z^{\{A\}} \setminus Z^{\{a\}}\right)
\end{aligned}
$$

by separating the global observed variables, X_G, into those who are direct children of $Z^{\{a\}}$, and those who are not. With the definition above and the ones given in Section 6.4, we can rewrite the KL-divergence as follows:

$$
D_{KL}\left[q_{\Phi^{\{a\}}}(Z)||\tilde{p}^{\{a\}}_{\Theta^{\{a\}}}(Z|X)\right] \tag{S2}
$$

$$
= \int_Z q_{\Phi^{\{a\}}}(Z)\log\left(\frac{q_{\Phi^{\{a\}}}(Z)}{\tilde{p}^{\{a\}}_{\Theta^{\{a\}}}(Z|X)}\right)dZ \tag{S3}
$$

$$
= \int_Z q_{\Phi^{\{a\}}}(Z)\log\left(\frac{q_{\Phi^{\{a\}}}\left(Z^{\{a\}}\right)\prod_{b\in A\setminus a} q_{\breve{\Phi}^{\{b\}}}\left(Z^{\{b\}}\right)}{\frac{p(X_G|Z^{\{A\}})}{\tilde{p}^{\{a\}}(X_G|X^{\{a\}})}p_{\Theta^{\{a\}},\mathrm{Pa}\breve{\Theta}(C^{\{a\}})}\left(Z^{\{a\}}|\mathrm{Pa}\breve{Z}\left(C^{\{a\}}\right), X^{\{a\}}\right)\prod_{b\in A\setminus a} q_{\breve{\Phi}^{\{b\}}}\left(Z^{\{b\}}\right)}\right)dZ \tag{S4}
$$

$$
= \int_Z q_{\Phi^{\{a\}}}(Z)\log\left(\frac{q_{\Phi^{\{a\}}}\left(Z^{\{a\}}\right)}{\frac{p(X_G|Z^{\{A\}})}{\tilde{p}^{\{a\}}(X_G|X^{\{a\}})}p_{\Theta^{\{a\}},\mathrm{Pa}\breve{\Theta}(C^{\{a\}})}\left(Z^{\{a\}}|\mathrm{Pa}\breve{Z}\left(C^{\{a\}}\right), X^{\{a\}}\right)}\right)dZ \tag{S5}
$$

$$
= \int_Z q_{\Phi^{\{a\}}}(Z)\log\left(\frac{q_{\Phi^{\{a\}}}\left(Z^{\{a\}}\right)}{\frac{p(X_G|Z^{\{A\}})}{\tilde{p}^{\{a\}}(X_G|X^{\{a\}})}\frac{p_{\Theta^{\{a\}},\mathrm{Pa}\breve{\Theta}(C^{\{a\}})}\left(Z^{\{a\}}, X^{\{a\}}|\mathrm{Pa}\breve{Z}(C^{\{a\}})\right)}{p_{\Theta^{\{a\}},\mathrm{Pa}\breve{\Theta}(C^{\{a\}})}\left(X^{\{a\}}|\mathrm{Pa}\breve{Z}(C^{\{a\}})\right)}}\right)dZ \tag{S6}
$$

$$= \int_Z q_{\Phi^{\{a\}}}(Z) \log \left(\frac{q_{\Phi^{\{a\}}}\left(Z^{\{a\}}\right)}{\frac{p(\mathrm{Ch}X_G(Z^{\{a\}})|\mathrm{Pa}Z(\mathrm{Ch}X_G(Z^{\{a\}})))p(X_G \setminus \mathrm{Ch}X_G(Z^{\{a\}})|Z^{\{A\}} \setminus Z^{\{a\}})}{\tilde{p}^{\{a\}}(X_G|X^{\{a\}})} \frac{p_{\Theta^{\{a\}},\mathrm{Pa}\breve{\Theta}\left(C^{\{a\}}\right)}\left(Z^{\{a\}}, X^{\{a\}}|\mathrm{Pa}\breve{Z}(C^{\{a\}})\right)}{p_{\Theta^{\{a\}},\mathrm{Pa}\breve{\Theta}\left(C^{\{a\}}\right)}\left(X^{\{a\}}|\mathrm{Pa}\breve{Z}(C^{\{a\}})\right)}} \right) dZ \tag{S7}$$

$$\begin{aligned}
&= \int_Z q_{\Phi^{\{a\}}}(Z) \log \left(\frac{q_{\Phi^{\{a\}}}\left(Z^{\{a\}}\right)}{p\left(\mathrm{Ch}X_G\left(Z^{\{a\}}\right)|\mathrm{Pa}Z\left(\mathrm{Ch}X_G\left(Z^{\{a\}}\right)\right)\right) p_{\Theta^{\{a\}},\mathrm{Pa}\breve{\Theta}(C^{\{a\}})}\left(Z^{\{a\}}, X^{\{a\}}|\mathrm{Pa}\breve{Z}\left(C^{\{a\}}\right)\right)} \right) dZ \\
&\quad + \int_Z q_{\Phi^{\{a\}}}(Z) \log \left(\tilde{p}^{\{a\}}(X_G|X^{\{a\}}) p_{\Theta^{\{a\}},\mathrm{Pa}\breve{\Theta}(C^{\{a\}})}\left(X^{\{a\}}|\mathrm{Pa}\breve{Z}\left(C^{\{a\}}\right)\right)\right) dZ \\
&\quad - \int_Z q_{\Phi^{\{a\}}}(Z) \log \left(p\left(X_G \setminus \mathrm{Ch}X_G\left(Z^{\{a\}}\right)|Z^{\{A\}} \setminus Z^{\{a\}}\right)\right) dZ
\end{aligned} \tag{S8}$$

$$\begin{aligned}
&= \underbrace{E_{Z\sim\tilde{q}^{\{a\}}_{\Phi^{\{a\}}}} \left[\log \left(\frac{q_{\Phi^{\{a\}}}\left(Z^{\{a\}}\right)}{p\left(\mathrm{Ch}X_G\left(Z^{\{a\}}\right)|\mathrm{Pa}Z\left(\mathrm{Ch}X_G\left(Z^{\{a\}}\right)\right)\right) p_{\Theta^{\{a\}},\mathrm{Pa}\breve{\Theta}(C^{\{a\}})}\left(Z^{\{a\}}, X^{\{a\}}|\mathrm{Pa}\breve{Z}\left(C^{\{a\}}\right)\right)} \right) \right]}_{-L^{\{a\}}_{KL}(\Theta^{\{a\}},\Phi^{\{a\}})} \\
&\quad + E_{Z\sim\tilde{q}^{\{a\}}_{\mathrm{Pa}\breve{Z}}} \left[\underbrace{\log \left(\tilde{p}^{\{a\}}(X_G|X^{\{a\}}) p_{\Theta^{\{a\}},\mathrm{Pa}\breve{\Theta}(C^{\{a\}})}\left(X^{\{a\}}|\mathrm{Pa}\breve{Z}\left(C^{\{a\}}\right)\right)\right)}_{LogEvd^{\{a\}}_{X_G,\ X^{\{a\}}}(\Theta^{\{a\}})} \right] \\
&\quad - \underbrace{E_{Z\sim\prod_{b\in A\setminus a} q_{\breve{\Phi}^{\{b\}}}(Z^{\{b\}})} \left[\log \left(p\left(X_G \setminus \mathrm{Ch}X_G\left(Z^{\{a\}}\right)|Z^{\{A\}} \setminus Z^{\{a\}}\right)\right)\right]}_{C}
\end{aligned} \tag{S9}$$

where

$$\tilde{q}^{\{a\}}_{\Phi^{\{a\}}} = q_{\Phi^{\{a\}}}\left(Z^{\{a\}}\right) \prod_{Z^{\{b\}} \in \mathrm{Pa}\breve{Z}(C^{\{a\}}) \cup \mathrm{Pa}\breve{Z}(\mathrm{Ch}X_G(Z^{\{a\}})) \setminus Z^{\{a\}}} q_{\breve{\Phi}^{\{b\}}}\left(Z^{\{b\}}\right)$$

is the joint variational distribution over the latent variables, $Z^{\{a\}}$, local to the a'th node collection, and the latent variables parent to the a'th node collection, $\mathrm{Pa}\breve{Z}\left(C^{\{a\}}\right)$, or having the same child global observed variables as the a'th node collection, $Z^{\{b\}} \in \mathrm{Pa}\breve{Z}\left(\mathrm{Ch}X_G\left(Z^{\{a\}}\right)\right) \setminus Z^{\{a\}}$. Furthermore,

$$\tilde{q}^{\{a\}}_{\mathrm{Pa}\breve{Z}} = \prod_{Z^{\{b\}} \in \mathrm{Pa}\breve{Z}(C^{\{a\}})} q_{\breve{\Phi}^{\{b\}}}\left(Z^{\{b\}}\right)$$

is the joint variational distribution over the latent variables parent to the a'th node collection, $\mathrm{Pa}\breve{Z}\left(C^{\{a\}}\right)$. Finally, $LogEvd^{\{a\}}_{X_G,\ X^{\{a\}}}\left(\Theta^{\{a\}}\right)$ denotes the joint log-evidence for X_G and $X^{\{a\}}$, and C is constant with respect to $\Theta^{\{a\}}, \Phi^{\{a\}}$. By simple rearranging terms we obtain the dual objective

$$L^{\{a\}}_{KL}\left(\Theta^{\{a\}}, \Phi^{\{a\}}\right) = E_{Z\sim\tilde{q}^{\{a\}}_{\mathrm{Pa}\breve{Z}}}\left[LogEvd^{\{a\}}_{X_G,\ X^{\{a\}}}\left(\Theta^{\{a\}}\right)\right] - D_{KL}\left[q_{\Phi^{\{a\}}}(Z)||\tilde{p}^{\{a\}}_{\Theta^{\{a\}}}(Z|X)\right] - C$$
